# Last-Generation Genome–Environment Associations Reveal the Genetic Basis of Heat Tolerance in Common Bean (*Phaseolus vulgaris* L.)

**DOI:** 10.3389/fgene.2019.00954

**Published:** 2019-11-22

**Authors:** Felipe López-Hernández, Andrés J. Cortés

**Affiliations:** ^1^Corporación Colombiana de Investigación Agropecuaria (Agrosavia) - CI La Selva, Rionegro, Colombia; ^2^Facultad de Ciencias – Grupo de Investigación en Sistemática Molecular, Universidad Nacional de Colombia - Sede Medellín, Medellín, Colombia; ^3^Facultad de Ciencias Agrarias - Departamento de Ciencias Forestales, Universidad Nacional de Colombia - Sede Medellín, Medellín, Colombia

**Keywords:** heat stress, local adaptation, genome-wide association studies (GWAS), environmental indices, SUPER, FarmCPU, BLINK

## Abstract

Genome–environment associations (GEAs) are a powerful strategy for the study of adaptive traits in wild plant populations, yet they still lack behind in the use of modern statistical methods as the ones suggested for genome-wide association studies (GWASs). In order to bridge this gap, we couple GEA with last-generation GWAS algorithms in common bean to identify novel sources of heat tolerance across naturally heterogeneous ecosystems. Common bean (*Phaseolus vulgaris* L.) is the most important legume for human consumption, and breeding it for resistance to heat stress is key because annual increases in atmospheric temperature are causing decreases in yield of up to 9% for every 1°C. A total of 78 geo-referenced wild accessions, spanning the two gene pools of common bean, were genotyped by sequencing (GBS), leading to the discovery of 23,373 single-nucleotide polymorphism (SNP) markers. Three indices of heat stress were developed for each accession and inputted in last-generation algorithms (*i.e.* SUPER, FarmCPU, and BLINK) to identify putative associated loci with the environmental heterogeneity in heat stress. Best-fit models revealed 120 significantly associated alleles distributed in all 11 common bean chromosomes. Flanking candidate genes were identified using 1-kb genomic windows centered in each associated SNP marker. Some of these genes were directly linked to heat-responsive pathways, such as the activation of heat shock proteins (*MED23*, *MED25*, *HSFB1*, *HSP40*, and *HSP20*). We also found protein domains related to thermostability in plants such as *S1* and *Zinc finger A20* and *AN1*. Other genes were related to biological processes that may correlate with plant tolerance to high temperature, such as time to flowering (*MED25*, *MBD9*, and *PAP*), germination and seedling development (*Pkinase_Tyr*, *Ankyrin-B*, and *Family Glicosil-hydrolase*), cell wall stability (*GAE6*), and signaling pathway of abiotic stress via abscisic acid (histone-like transcription factors *NFYB* and *phospholipase C*) and auxin (*Auxin response factor* and *AUX_IAA*). This work offers putative associated loci for marker-assisted and genomic selection for heat tolerance in common bean. It also demonstrates that it is feasible to identify genome-wide environmental associations with modest sample sizes by using a combination of various carefully chosen environmental indices and last-generation GWAS algorithms.

## Introduction

Exploring the genetic basis of adaptive traits in wild plant populations has been accelerated by modern genomic strategies such as genome–phenotype [genome-wide association study (GWAS)] and genome–environment association (GEA) studies (Frank et al., 2016). GEA commonly associates single-nucleotide polymorphisms (SNPs) and environmental variables based on the accessions’ sampling site in order to infer adaptation to abiotic stress. Genotyping by sequencing (GBS) has in turn been revealed as one of the best methods for GEA due to its potential to discover a considerable amount of SNP markers throughout the genome. For instance, coupling GEA and GBS recently allowed identifying adaptive variation for drought tolerance (Cortés and Blair, 2018). However, despite the fact that the GEA framework uses the latest genomic tools available, it has not yet taken full advantage of newer and more promising statistical approaches to detect genomic signatures of environmental adaptation while controlling for confounding effects.

GEA studies often rely on GWAS models, which typically couple mixed linear models (MLMs) (Zhang et al., 2010) with kinship and population structure analyses in order to correct for false positives. Yet new GWAS algorithms have recently been developed to gain statistical power to detect associated markers, increase efficiency, and decrease computational complexity (Wang et al., 2014b). The strategy to reconstruct the kinship matrix is the most relevant difference between recent methods of individual marker tests such as Factored Spectrally Transformed Linear Mixed Model (FaST-LMM-Select), Compressed MLM (CMLM) (Li et al., 2014), and Settlement of MLM Under Progressively Exclusive Relationship (SUPER), the latter being the most statistically powerful (Wang et al., 2014b; Liu et al., 2016). SUPER drastically reduces the amount of genetic markers used to infer kinship relationships by dividing the SNP dataset into *bins* (Wang et al., 2014b). Most influential *bins*, known as pseudo-nucleotides of quantitative rank underlying the phenotype (PseudoQTNs), are then optimized in size and number using maximum likelihood and linkage disequilibrium (LD). On the contrary, FaST-LMM-Select chooses SNPs to infer kinship relationships based only on a physical distance criterion, while CMLM uses kinship estimates between pairs of groups clustered based on their kinship value in order to reduce the size of the fixed effect and increase the computational power. Tests of multiple loci such as the multi-locus mixed model (MLMM) (Segura et al., 2012) have been developed, too. Both strategies, individual markers (CMLM, FaST-LMM-Select, and SUPER) and multiple loci (MLMM) tests, effectively control the false-positive rate. Yet these algorithms have a higher rate of false negatives after the partition imposed on the SNP dataset to recreate the kinship matrix.

Alternative methods such as Fixed and random model Circulating Probability Unification (FarmCPU) (Liu et al., 2016) and Bayesian-information and Linkage-disequilibrium Iteratively Nested Keyway (BLINK) (Huang et al., 2019) have been developed to control both the false-positive rate and the confounding variable that disfavors the real associations. FarmCPU and BLINK divide a typical MLMM into two parts that are used iteratively, a fixed effect model (FEM) and a random effect model (REM). BLINK replaces restricted maximum likelihood (REML) in FarmCPU’s REM with Bayesian information criteria (BIC) in a FEM. Additionally, BLINK uses LD information to replace the bin method. SUPER, FarmCPU, and BLINK can therefore be considered last-generation GWAS models. These powerful algorithms, already tested for conventional GWAS, are promising to identify adaptive loci under a GEA framework.

In turn, the potential of GEA studies to identify new sources of tolerance to abiotic stresses is undeniable (Cortés and Blair, 2018) and could aid the study of the genetic adaptation to adverse conditions that have not previously been approached from a GEA perspective, which is the case of heat stress (HS). Annual increases in atmospheric average temperature have been responsible for yield losses of 9% for every 1°C across the vast majority of agricultural species. This situation is likely to worsen as by 2100 global average temperature is estimated to be 3°C above the present value (Abrol and Ingram, 1996), jeopardizing worldwide yields.

Common bean (*Phaseolus vulgaris* L.), a not perennial (Gentry, 1969), is one of the most produced legumes with ∼27 million tons worldwide, China and America being the main producers (FAO, 2018), yet tolerance to HS is generally low in this species. Beans are nutritionally rich due to their high content of proteins, folic acid, iron, dietary fiber, and complex carbohydrates and constitute a main alimentary supply for communities in Latin America, Africa, and Asia (Sgarbieri and Whitaker, 1982; Pachico, 1993). Since these regions are also highly vulnerable to HS, increased atmospheric average temperature would impact not only yields in small-scale farms but also human nutrient intake *via* common bean (Jones, 1999). Most common bean varieties used by farmers are better adapted to regions of medium to high elevations or to sowing times during the colder seasons in tropical areas (Porch and Jahn, 2001). Some authors have reported optimal temperatures between 18°C and 20°C (Wantanbe, 1953; Qi et al., 1998; Porch, 2006; Rosas et al., 2000) for the cultivation of this legume. The reproductive phase is the most sensitive phenological stage to HS, with temperatures above 28°C to 32°C (Gonçalves et al., 1997; Caramori et al., 2001; Silva et al., 2007; Rainey and Griffiths, 2019) decreasing the number of pods and seeds and therefore reducing yield (Weaver and Timm, 1988; Monterroso and Wien, 2019). In order to compensate for yield losses due to low tolerance of cultivated common bean to high temperatures, a prompt characterization of the genetic sources of HS tolerance in wild populations is needed.

Nowadays, there is a lack of knowledge on how the most recent GWAS models work under a GEA paradigm. Additionally, there is an urgent need to identify loci linked to HS tolerance in wild common bean germplasm collections, which would aid the development of common bean varieties resistant to high temperatures. Therefore, for this study, we set the following objectives: (1) synthetize environmental variables in order to estimate HS tolerance in wild common bean germplasm collections, which would allow identifying tolerant accessions; (2) explore the utility of the most promising modern GWAS models (CMLM, SUPER, FarmCPU, and BLINK) for GEA studies; and (3) implement GEA models with last-generation GWAS algorithms in order to capture adaptive genetic variation to HS, candidate to be integrated into common bean breeding programs. This first exploration of the environmental adaptation of wild common bean to HS will ultimately offer putative associated loci for marker-assisted and genomic selection strategies by using a combination of various well-chosen environmental indices and last-generation GWAS algorithms, while testing the utility of the latter under a GEA paradigm.

## Materials and Methods

### Plant Material and GBS

The present work was developed with a total of 78 accessions of wild common bean. All genotypes were transferred by the Genetic Resources Unit of the International Center for Tropical Agriculture (CIAT) and are conserved under the genetic resources treaty of the Food and Agriculture Organization of the United Nations (FAO collection). The accessions are a representative sample of groups of genes and races, the selection being based on core collections for wild bean samples according to Tohme et al. (1996). Despite adaptation to environmental stress conditions evolved differently in the two gene pools of common bean (Soltani et al., 2017; Soltani et al., 2018; Oladzad et al., 2019), we carried out the GEA models including both gene pools in order to maximize the statistical power to detect significantly associated markers by increasing (1) the number of wild accessions and (2) the environmental contrast (Mesoamerican environments of wild common bean typically experience more heat events than Andean environments, **Figure S8**). Georeferencing was provided by the Genetic Resources Unit at CIAT (**Table S1**).^1^

Processing of plant material, genomic DNA extraction, GBS library preparation using the *Apek1* enzyme (Cortés and Blair, 2018), and sequencing and bioinformatic processing for the 78 accessions were carried out as described by Cortés and Blair (2018), following Elshire et al. (2011) and Bradbury et al. (2007) and using as reference genome the common bean assembly (Schmutz et al., 2014). SNP markers with missing data that exceeded 20% or frequency of the minor allele (MAF) that did not exceed 5% were excluded from the GEA dataset in the 78 genotyped accessions in order to finally obtain a matrix of 23,373 SNP markers with an average depth of 13.6 X.

### Compilation of Bioclimatic Data and HS Indices

In order to estimate heat tolerance for wild common bean, we extracted from the WorldClim^2^ database, at a 2.5-min resolution, environmental variables using the georeferencing of each accession. A total of six bioclimatic variables, putatively related with HS, were considered, as follows: BIO1 = annual mean temperature, BIO5 = maximum temperature of warmer month, BIO8 = mean temperature of the wettest quarter, BIO9 = mean temperature of the driest quarter, BIO10 = mean temperature of the warmest 4-month period, and *T* *_j_* = average of absolute maximum temperature during the reproductive phase. Extraction was carried out using the *dismo* package of R v.3.4.4 (R Core Team). Historical temperature values were obtained as monthly averages from 1970 to 2000. Values of each bioclimatic variable were adjusted for the year 2000 according to the average annual increase in temperature for each hemisphere, using the following expressions (Trenberth et al., 2007):

(1) $$T_{2000}=\text{Ti}+\left(2000-i\right)\times 0.031675\;[{}^{\circ}C]\text{ for the Northern Hemisphere}$$

(2) $$T_{2000}=\mathrm{Ti}+\left(2000-i\right)\times 0.01325\;[{}^{\circ}C]\text{ for the Southern Hemisphere}$$

where *i* is the year of collection of each accession, *Ti* is the bioclimatic variable for the year when the accession was collected, and *T*_2000_ is the value of each bioclimatic variable for the year 2000.

We generated three indices based on environmental data from wild common bean accessions in order to understand natural adaptation to high temperatures and identify associated genetic markers. The first index was built using the evapotranspiration model from (Thornthwaite, 1948), which contained an expression for monthly heat index, heat index Thornthwaite (HIT), as follows (equation 3):

(3) $$\mathrm{HI}T_{\text{original}}=\sum_{i=1}^{k}\frac{T_{m}^{1.514}}{5}$$

For all *T*_m_ > 0, *T*_m_ is the average mean monthly temperature in any phenological stage of the plant, and *k* the number of months.

This index (*HIT*_original_) uses average temperature (*T*_m_) and not maximum temperature (*T* *_j_*), despite the latter being more informative for HS events. Thus, we used two adjustments to refine *HIT*_original_. First, we used the absolute maximum temperature instead of the average temperature. Second, we narrowed the window of temperatures only across the reproductive phase (*T* *_j_*), during which plants are most sensitive to HS events (Rainey and Griffiths, 2019). Since seeds were collected for each accession as part of the original sampling, the reproductive phase has an approximate duration of 2 months prior to the month when sampling took place. The modified *HIT*_original_ index was expressed through the following equation:

(4) $$\mathrm{HIT}=\sum_{i=1}^{2}\frac{T_{j}^{1.514}}{5}$$

For all *T* *_j_* > 0, *T* *_j_* is the average of absolute maximum temperature during the reproductive phase and *i* is the month within that phase.

On the other hand, we built a second index of HS, heat stress index (HSI), as detailed in equation 5. This index is based on the temperature threshold during the reproductive phase (*T*_max_ = 28–32°C) above which common bean exhibits low grain yields (Gonçalves et al., 1997; Caramori et al., 2001; Silva et al., 2007; Rainey and Griffiths, 2019). Therefore, this suggested HS index compares *T*_max_ = 30°C and the maximum temperature during the reproductive phase *T* *_j_* adjusted for the year 2000.

(5) $$\mathrm{HSI}=\left(\frac{T_{j}-30}{30}\right)\times 100;-100\leq \mathrm{HSI}\leq 100$$

Finally, the first main principal component of all six bioclimatic variables explained 94.37% of the overall variance and was chosen as a third index of HS (hereinafter referred to as PCA1). Using all three indices aims characterizing different components of the adaptation to HS. Two important assumptions of these HS indices should be noted. First, poorly adapted genotypes are inexistent because the distribution of accessions in the study areas is assumed to be in equilibrium with the niche requirements (Forester et al., 2016). Second, it is assumed that HS indices are stable over the years, since they are based on climatic data averaged over three decades. Ecological balance and stability of these HS indices are a prerequisite for GEA analysis (Cortés et al., 2013; Cortés and Blair, 2018). Since normality is also required for GWAS-type models, normality of each bioclimatic variable was verified using the skewness, kurtosis, and Shapiro–Wilk statistics (*P* ≥ 0.05) using the *agricolae* package (De Mendiburu, 2014) in R v.3.4.4 (R Core Team). Dispersion diagrams, as well as Pearson (*r*) and Spearman (*ρ*) correlations, were made among all bioclimatic variables and HS indices in R v.3.4.4 (R Core Team).

### Analysis of Kinship and Population Structure

Using the panel of 23,373 SNP markers, we estimated random and fixed effects in order to reduce the rate of false positives of each GEA model (*i.e.* MLM, CMLM, SUPER, FarmCPU, and BLINK). Random effects accounted for kinship relationships, while fixed effects accounted for population structure. Kinship was built in different ways according to the peculiarities of each algorithm. The MLM used a kinship matrix computed across all markers using the Loiselle, VanRaden, and EMMA methods available in the *GAPIT* package (Tang et al., 2016) of R v.3.4.4 (R Core Team). As an exploratory phase, we tested the power of these three different methods in capturing random effects in a GEA with MLM models. MLM models were selected for this purpose because they consider all 23,373 SNP markers. MLM models were designed using the combination of all three HS indices as response variable “I” (HIT, his, and PCA1), two population stratification methods as fixed effects “Q” (PC and TESS3), and three kinship methods as random effects “K” (Loiselle, VanRaden, and EMMA) for a total of 18 MLM models (3I × 2Q × 3K). Among all 18 MLM models, those that used the EMMA algorithm to reconstruct the kinship matrix were the most powerful. Thus, the following GEA models only considered the EMMA algorithm.

Based on this exploratory phase, only the EMMA algorithm was implemented for the reconstruction of the kinship relationships in the improved MLM algorithms (*i.e.* CMLM) and the last-generation GWAS models (*i.e.* SUPER, FarmCPU, and BLINK), each of which had different criteria for sub-setting the SNP dataset (PseudoQTNs) according to their specifications (Wang et al., 2014b; Liu et al., 2016; Huang et al., 2019).

Population stratification was explored using two strategies. First, a traditional molecular principal component analysis (hereinafter referred to as PC) was carried out in TASSEL v.5 (Bradbury et al., 2007). Second, spatial population structure was reconstructed using *TESS3* (Caye et al., 2016) as implemented in R v.3.4.4 (R Core Team). *TESS3* is a novel package that infers population structure from genotypic and geographical information. The optimum number of ancestral populations (*K*) was determined using a cross-entropy method implemented with the *snmf* function in the *LEA* package (Frichot and François, 2015) of R v.3.4.4 (R Core Team). The *snmf* algorithm was executed with 1,000 repetitions and a fluctuating K value from 2 to 10. The cross-entropy inference was further improved by exploring the percentage of masked genotypes at thresholds of 5% and 20%, as suggested by Frichot and François (2015) and Ariani et al. (2018), respectively. Results of population stratification were compared explicitly with previous studies carried out in wild common bean by Ariani et al. (2018). We selected a clustering coefficient (*Q*) cutoff of ≥0.7, following Ariani et al. (2018) and Bitocchi et al. (2012), for assigning genotypes to subpopulations.

### Identification of Loci Associated With HS Indices

After the exploratory phase with 18 MLM models, we built 30 GEA models using improved MLM (CMLM) and last-generation GWAS (*i.e.* SUPER, FarmCPU, and BLINK) algorithms to explore single-marker associations. The improved MLM and last-generation GWAS algorithms increase the statistical power while better controlling the false-positive rate. FarmCPU and BLINK are particularly powerful at further controlling the false-negative rate (Huang et al., 2019). GEA models were obtained from the combination of all three HS indices as response variable “I” (HIT, HSI, and PCA1), two population stratification methods as fixed effects “Q” (PC and TESS3), and a unique kinship method as random effect “K” (EMMA with PseudoQTNs) for a total of 30 GEA models constructed by means of one improved MLM algorithm (CMLM) and three last-generation GWAS algorithms (SUPER, FarmCPU, and BLINK). GEA models considered a total of six CMLM models (3I × 2Q × 1K), six FarmCPU models (3I × 2Q × 1K), six BLINK models (3I × 2Q × 1K), and 12 SUPER models. SUPER models were initially implemented as suggested by Wang et al. (2014b) in order to be computationally efficient, yet expecting the same statistical power as any MLM and CMLM models. To overcome this issue, these first-stage SUPER models were coupled with the MLM and CMLM algorithms for a total of 12 second-stage SUPER models (3I × 2Q × 1K × 1 first-stage GWAS algorithm × 2 second-stage GWAS algorithms).

Models were abbreviated as follows: I_M-Fc-Rc_, where “I” refers to the HS index, “M” is the GWAS model family, and “Fc” and “Rc” are the algorithms used to reconstruct the fixed and random covariates, respectively. For example, the model HIT_FARMCPU-TESS3-EMMA_ used HIT as the HS index, FarmCPU as the GWAS method, *TESS3*’s inference as a fixed covariate, and EMMA’s kinship as a random covariate. This nomenclature was modified to account for the SUPER algorithm since it employed two different GWAS models in the first and last steps. The first step always used a GLM model, but the last step used a MLM or CMLM model. Therefore, SUPER models were marked as I_SUPER(M)-Fc-Rc_, where “M” is the model used in the last step (MLM or CMLM) (**Table S2**).

In order to choose the optimal GEA models, we drew Q–Q and Manhattan diagrams of the *P*-values with customized R scripts and used these diagrams to evaluate the rate of false positives. Highly significant associations were determined using a Bonferroni correction of *P*-values at an *α* = 0.05, which led to a significance threshold of 2.14 × 10^−6^ or −log_10_2.14 × 10^−6^ = 5.67 for CMLM models (2,373 effective SNP markers), 2.13 × 10^−6^ or −log_10_2.13 × 10^−6^ = 5.67 for SUPER models (23,421 effective SNP markers), and 5.89 × 10^−6^ or −log_10_5.89 × 10^−6^ = 5.23 for FarmCPU and BLINK models (8,494 effective SNP markers). Therefore, we used the Bonferroni threshold in order to evaluate the rate of false positives by visual interpretation of the Q–Q plots. In addition, a relax threshold of −log_10_ *P*-value = 4, as previously suggested (Pasam et al., 2012; Soltani et al., 2017; Soltani et al., 2018; Oladzad et al., 2019), was used only in the exploratory phase with 18 MLM models in order to identify weaker associations, since it is documented that the Bonferroni threshold is very restrictive or conservative in MLMs (Joo et al., 2016).

### Identification of Candidate Genes

Putative candidate genes were identified by inspecting conservative flanking sections of 1 kb around each associated locus from all GEA models. Flanking sections were captured using the common bean reference genome v2.1 (Schmutz et al., 2014) and the *PhytoMine* and *BioMart* tools from the Phytozome v.12.3 platform.^3^ Identified genes were further annotated using the GO,^4^ PFAM,^5^ PANTHER,^6^ KEGG,^7^ and UniProt^8^ databases by means of Phytozome (see note C). Authors such as Oladzad et al. (2019) and Soltani et al. (2017; 2018) have suggested a genomic window to look for flanking genes of ∼100 kb in common bean. On the other hand, LD in wild common bean, measured as marker correlation *R* ^2^, was reported to decay to 0.8 per every 81 kb (Rossi et al., 2009). Thus, we further explored a genomic window of 81 kb (40.5 kb upstream to 40.5 kb downstream of the significantly associated SNP markers) using the common bean reference genome v2.1 and the annotation tools as described above.

## Results

Among the entire set of 78 wild common bean accessions, we identified five accessions (G2648, G23511A, G13094, G12869, and G11071) putatively tolerant to HS based on three different bioclimatic indices (HIT, HSI, and PCA1). Incorporating these indices as response variables in GEA models led to 18 traditional MLM models that used three contrasting kinship reconstruction methods and 30 improved traditional mixed (*i.e.* ECLMLM) and last-generation GWAS models (*i.e.* SUPER, FarmCPU, and BLINK) that only used the EMMA algorithm for kinship reconstruction. None of the improved traditional mixed models yielded significant results. On the other hand, 15 last-generation GWAS models increased the statistical power to detect 120 significant associations in a GEA framework. A joint inference across these models and the three indices allowed having a more comprehensive understanding of the adaptive landscape and genetic architecture of heat tolerance. We recovered 22 genes, flanking 24 SNP markers, previously reported as candidates for heat tolerance (Wang et al., 2004; Ikeda et al., 2011; Lopes-Caitar et al., 2013; Oladzad et al., 2019; Soltani et al., 2019) and involved in the activation of heat shock proteins (HSPs), protein domains related to thermostability in plants and signaling pathways of abiotic stress via abscisic acid and auxin. These allelic variants require further validation and are ideal to be incorporated into common bean breeding programs for resistance to high temperatures.

### Each Bioclimatic Index Captured a Different Component of HS

The three HS indices captured different facets of HS. All six bioclimatic variables (annual average temperature, maximum temperature of the warmest month, average temperature of the wettest quarter, average temperature of the driest quarter, average temperature of the warmest quarter, and average of the absolute maximum temperature of the reproductive phase) and three HS indices (HIT, HSI, and PCA1) exhibited a normal behavior (Shapiro–Wilk *P* ≥ 0.05, **Figure S1**). HIT and PCA1 presented a positive bias with a skewness statistics of 0.160 and 0.271, respectively. On the other hand, HSI had a negative skewness with a skewness value of −0.166. All three HS indices allowed us to approximate the same HS event by different strategies. If different indices had distinct skewness values, contrasting extreme values described different facets of the HS event (**Figure S1**). Correlation coefficients estimated by Pearson (*r*) and Spearman (*ρ*) methods respectively ranged from 0.82 to 1 and from 0.78 to 1 among all bioclimatic variables and the HIT and HSI indices. The index built with the PCA1 had a negative correlation with all six bioclimatic variables and the other two HS indices (**Figure S2**) with Pearson (*r*) and Spearman correlation coefficients (*ρ*) ranging from −0.92 to −0.99 and −0.94 to −0.99, respectively. Therefore, despite differences in the extreme values, there is correspondence among all six bioclimatic variables and the three indices. Normality, together with the assumptions of stability over time and genotype–ecological niche equilibrium, makes these three HS indices suitable as response variables in GWAS models within a GEA framework aiming to capture various components of HS.

### All 23,373 SNP Markers Recovered Six Subpopulations

**Figure 1:**
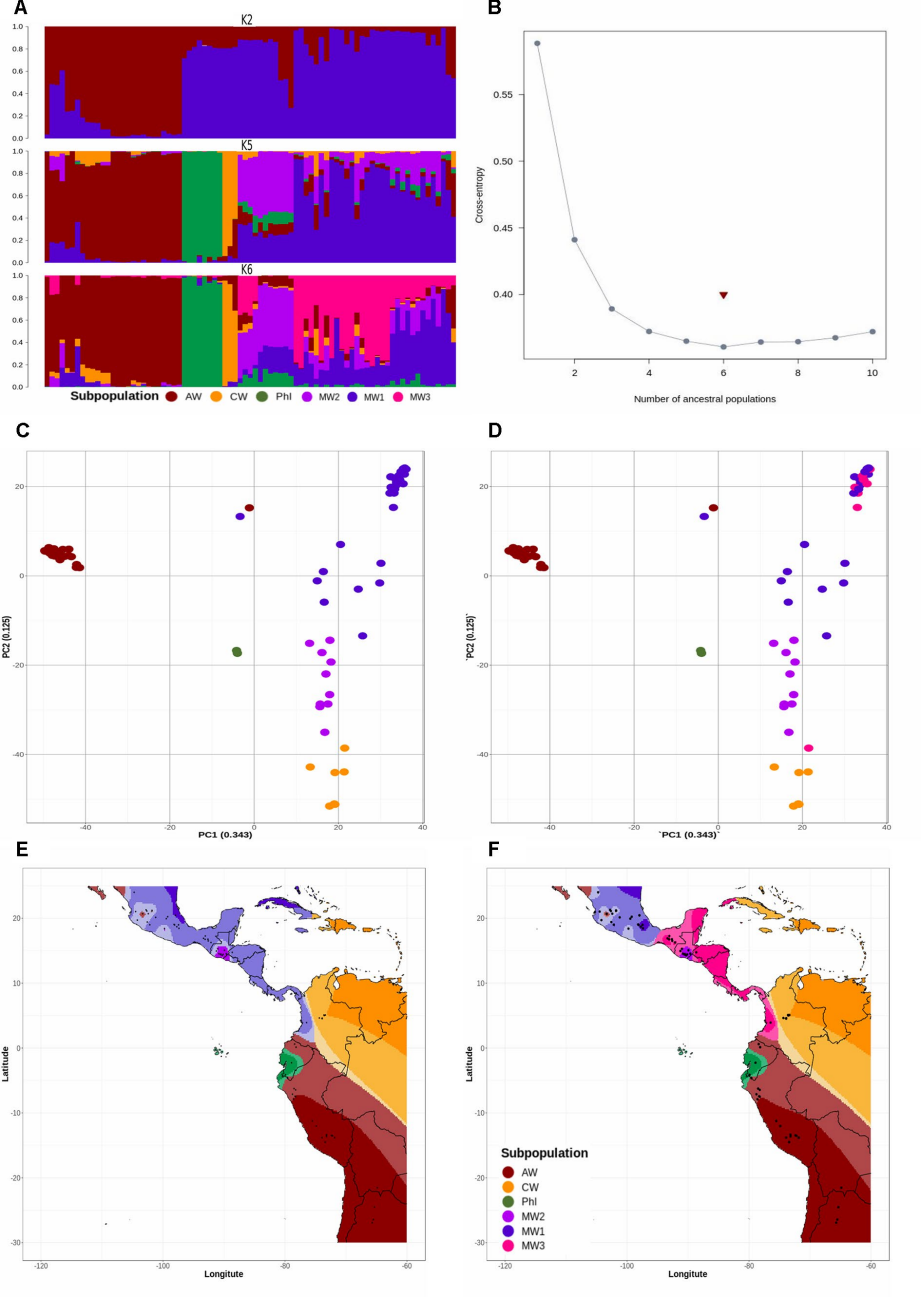
**(A)** Spatial population clustering and ancestry coefficients estimated with TESS3 using the number of gene pools (*K* = 2), the number of subpopulations suggested by other studies (*K* = 5), and the best number of subpopulations suggested by cross-entropy validation test (*K* = 6). The genotypes are sorted by latitude from northern Argentina to Mesoamerica. The subpopulations are MW1 (Mesoamerican Wild 1), MW2 (Mesoamerican Wild 2), MW3 (Mesoamerican Wild 3), PhI (Northern Peru-Ecuador Wild), AW (Andean Wild), and CW (Colombian Wild), colored in blue, purple, pink, green, red, and yellow, respectively.** (B)** Cross-entropy plot when the number of cluster (*K*) ranges between 1 and 10. The snmf algorithm was executed with 1,000 repetitions and a fluctuating *K* value from 2 to 10. The cross-entropy inference was further improved by exploring the percentage of masked genotypes at thresholds of 5% and 20%. **(C**, **D)** Population structure revealed by a molecular principal component analysis based on 23,373 SNP markers, using number of subpopulations *K* = 5 **(C)** and *K* = 6 **(D)**. Subpopulations are colored as in **(A)**. The percentage of explained variation by each axis is shown within parenthesis in the label of the corresponding axis. **(E**, **F)** Spatial interpolation of population ancestry coefficients across the geographic distribution of the genotypes analyzed. Subpopulations are colored as in **(A)**.

Population structure as revealed by a PC (molecular PCA) analysis with 23,373 SNP markers suggested a total of six subpopulations (**Figure 1**). Also, cross-entropy validation implemented in *TESS3* with the same markers suggested an optimum of six subpopulations from Mesoamerica to northern Argentina (**Figure 1B**). Both methods, *TESS3* and PC, suggested six subpopulations: MW1 (Mesoamerican Wild 1), MW2 (Mesoamerican Wild 2), MW3 (Mesoamerican Wild 3), PhI (Northern Peru–Ecuador Wild), AW (Andean Wild), and CW (Colombian Wild) (**Figures 1D–F**). When we looked at the five subpopulations partition suggested by Ariani et al. (2018) based on following previous works 19,126 SNP markers flanking the *Cvi*AII restriction site, we did not recover Ariani’s MW3 (**Figures 1C–E**), but instead the new subpopulation CW reappeared in both analyses (*TESS3* and PC).

### EMMA Algorithm Was More Powerful at Reconstructing Kinship Relationships

**Figure 2:**
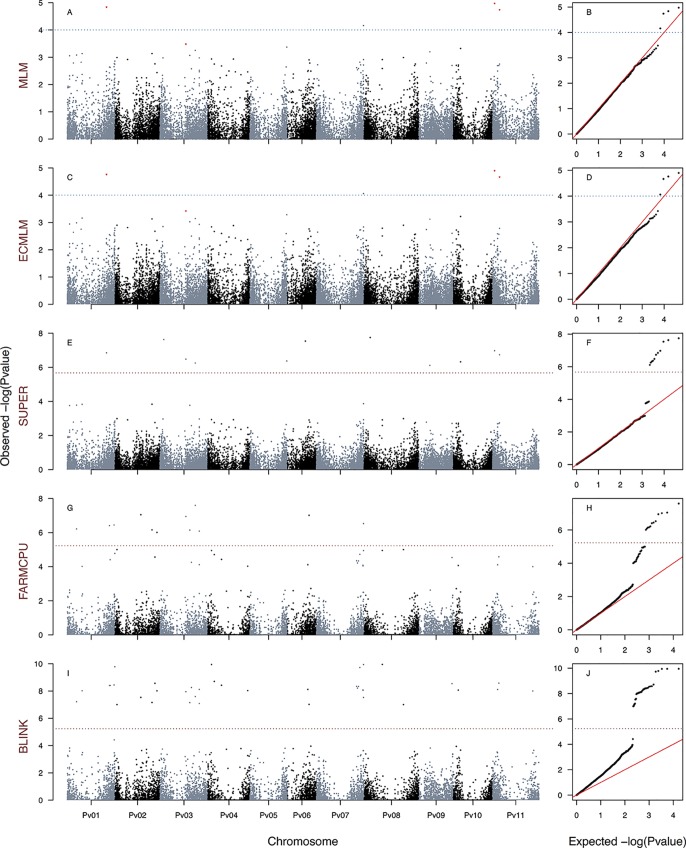
Manhattan and Q–Q plots of the optimum genome–environment association (GEA) analysis for heat tolerance in 78 common bean accessions based on 23,373 SNP markers, using the HSI index (equation 4). The Manhattan and Q–Q plots are generated according to traditional MLM algorithms, compressed MLM algorithms, and last-generation GWAS algorithms (SUPER, FarmCPU, and BLINK) using kinship matrix as a random effect by the EMMA algorithm and the first six principal components (**Figure 1D**) as a fixed effect. These models are HSI_MLM-PC-EMMA_ **(A**, **B)**, HSI_CMLM-PC-EMMA_ **(C**, **D)**, HSI_SUPER-PC-EMMA_ **(E**, **F)**, HSI_FARMCPU-PC-EMMA_ **(G**, **H)**, and HSI_BLINK-PC-EMMA_ **(I**, **J**). The red dashed horizontal line marks the *P*-value threshold after Bonferroni correction for multiple comparisons. The blue dashed horizontal line marks the lax *P*-value threshold. Black and blue colors highlight different common bean (Pv) chromosomes.

**Figure 3:**
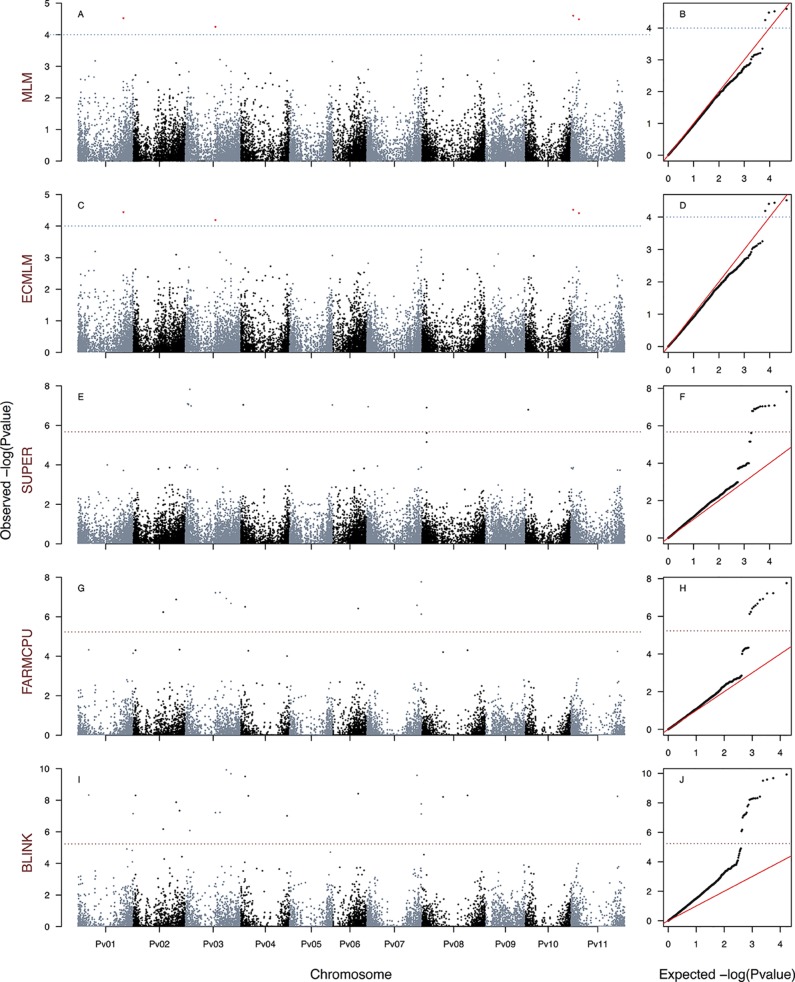
Manhattan and Q–Q plots of the optimum genome–environment association (GEA) analysis for heat tolerance in 78 common bean accessions based on 23,373 SNP markers, using the HIT index (equation 5). The Manhattan and Q–Q plots are generated according to traditional MLM algorithms, compressed MLM algorithms, and last-generation GWAS algorithms (SUPER, FarmCPU, and BLINK) using kinship matrix as a random effect by the EMMA algorithm and the first six principal components (**Figure 1D**) as a fixed effect. These models are HIT_MLM-PC-EMMA_ **(A**, **B)**, HIT_CMLM-PC-EMMA_ **(C**, **D)**, HIT_SUPER-PC-EMMA_ **(E**, **F)**, HIT_FARMCPU-PC-EMMA_ **(G**, **H)**, and HIT_BLINK-PC-EMMA_ **(I**, **J)**. The red dashed horizontal line marks the *P*-value threshold after Bonferroni correction for multiple comparisons. The blue dashed horizontal line marks the lax *P*-value threshold. Black and blue colors highlight different common bean (Pv) chromosomes.

**Figure 4:**
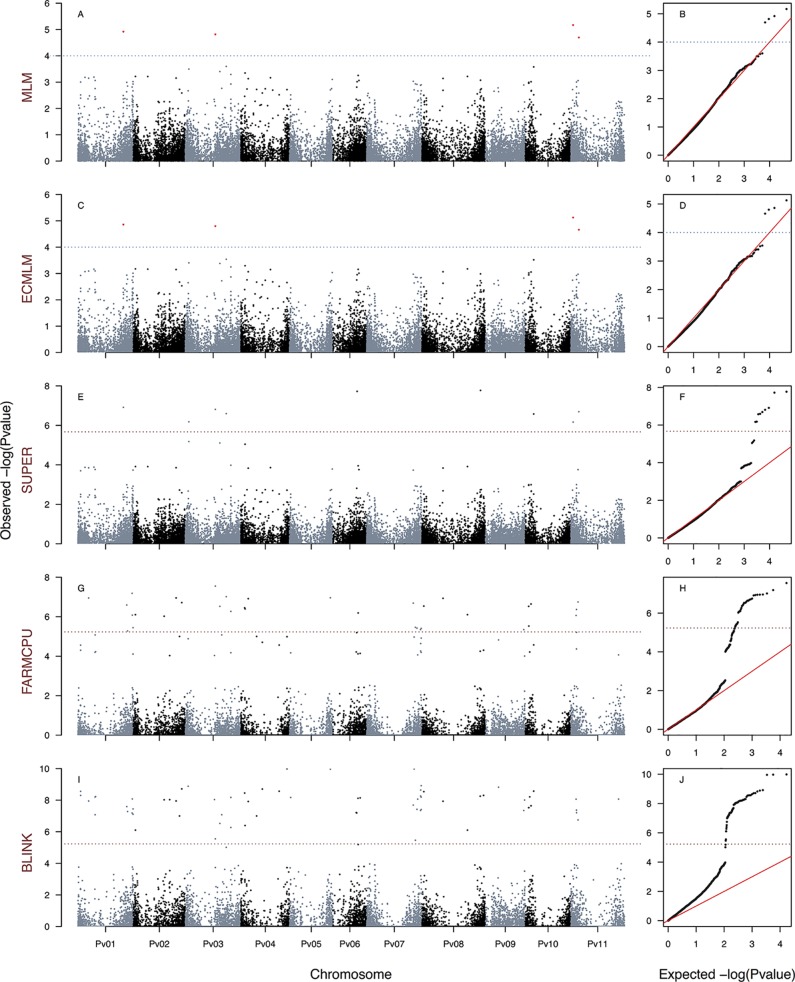
Manhattan and Q–Q plots of the optimum genome–environment association (GEA) analysis for heat tolerance in 78 common bean accessions based on 23,373 SNP markers, using the PCA1 index. The Manhattan and Q–Q plots are generated according to traditional MLM algorithms, compressed MLM algorithms, and last-generation GWAS algorithms (SUPER, FarmCPU, and BLINK) using kinship matrix as a random effect by the EMMA algorithm and the first six principal components (**Figure 1D**) as a fixed effect. These models are PCA1_MLM-PC-EMMA_ **(A**, **B)**, PCA1_CMLM-PC-EMMA_ **(C**, **D)**, PCA1_SUPER-PC-EMMA_ **(E**, **F)**, PCA1_FARMCPU-PC-EMMA_ **(G**, **H)**, and PCA1_BLINK-PC-EMMA_ **(I**, **J)**. The red dashed horizontal line marks the *P*-value threshold after Bonferroni correction for multiple comparisons. The blue dashed horizontal line marks the lax *P*-value threshold. Black and blue colors highlight different common bean (Pv) chromosomes.

As an exploratory phase, we built 18 traditional MLM models incorporating as random effects kinship matrices estimated with the Loiselle, VanRaden, and EMMA algorithms and as fixed effects estimates from TESS3 and PC (molecular PCA) algorithms across all 23,373 SNP markers. The three kinship algorithms were congruent among them and with the inferred population structure, revealing the typical Mesoamerican–Andean gene pool split (**Figure S3**). None of these 18 traditional MLMs recovered associated markers at a Bonferroni threshold of −log_10_ *P*-value = 5.67 (**Figures 2**–**4A, B**, **S4**, **S5A–L**, and **S6A–F**). Three loci systematically crossed the lax threshold of −log_10_ *P*-value = 4. They were on chromosomes Pv01 (S1_42870591) and Pv11 (S1_466464831 and S1_471851336) in all 18 traditional MLM models (**Figures 2A, B**, **3A, B**, **4A, B**, **S4A–L**, **S5A–L**, and **S6A–F**). Three of the models built with the EMMA algorithm (HIT_MLM-PC-EMMA_, HSI_MLM-PC-EMMA_, and PCA1_MLM-PC-EMMA_) further identified three other alleles that crossed the lax threshold with greater significance (**Figures 2**–**4A, B**). Thus, the EMMA-based kinship matrix was defined as the random effect for the 30 improved traditional mixed and last-generation GWAS models.

### A Total of 120 Loci in 15 Models Were Associated With the Three HS Bioclimatic Indices

We generated a total of 30 GEA models by implementing the algorithms CMLM (six models), SUPER (12 models), FarmCPU (six models), and BLINK (six models) using three HS indices as response variables, two methods of population stratification (PC and TESS3) as a fixed effect, and kinship reconstruction using the EMMA algorithm as a random effect. None of the six CMLM (**Figures 2**–**4C, D** and **S6G–L**) models yielded associated markers at a Bonferroni threshold of −log_10_ *P*-value = 5.67. However, at a lax threshold of −log_10_ *P*-value = 4, these CMLM models captured the same three associated loci identified by the 18 traditional MLMs. Three CMLM models that used the PCA1 as a covariable (HIT_CMLM-PC-EMMA_, HSI_CMLM-PC-EMMA_, and PCA1_CMLM-PC-EMMA_) captured, at a lax threshold, one additional associated locus each (**Figures 2**–**4C, D**).

**Figure 5:**
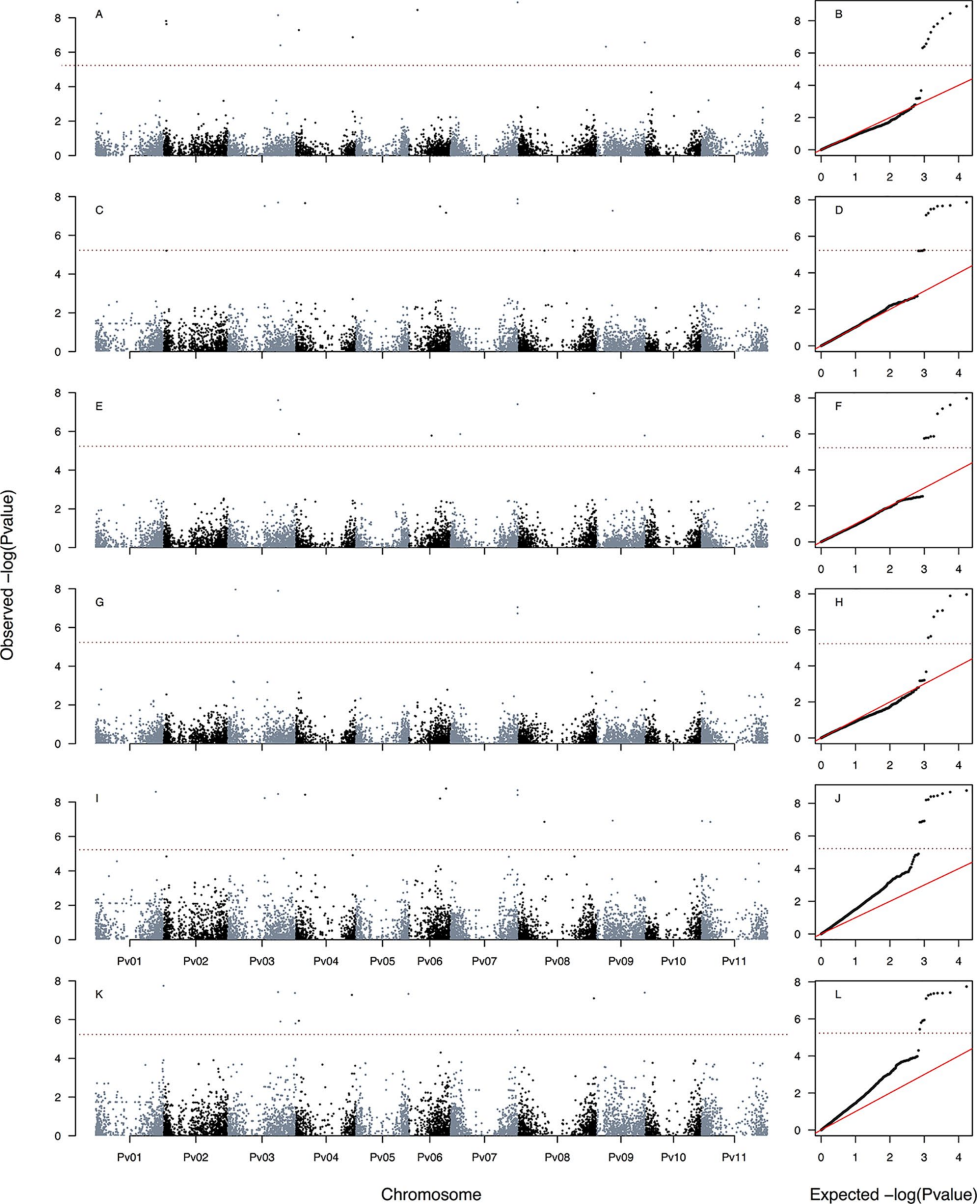
Manhattan and Q–Q plots of the optimum genome–environment association (GEA) analysis for heat tolerance in 78 common bean accessions based on 23,373 SNP markers according to last-generation GWAS algorithms FarmCPU and BLINK. The covariates used in these six models provided are kinship matrix as a random effect using EMMA algorithm and the population structure as fixed effect using TESS3 (**Figure 1F**). These last-generation GWAS models are HSI_FARMCPU-TESS3-EMMA_ **(A**, **B)**, HIT_FARMCPU-TESS3-EMMA_ **(C**, **D)**, PCA1_FARMCPU-TESS3-EMMA_ (**E**, **F**), HSI_BLINK-TESS3-EMMA_ **(G**, **H)**, HIT_BLINK-TESS3-EMMA_ **(I**, **J)**, and PCA1_BLINK-TESS3-EMMA_ **(K**, **L)**. The red dashed horizontal line marks the *P*-value threshold after Bonferroni correction for multiple comparisons. Black and blue colors highlight different common bean (Pv) chromosomes.

**Figure 6:**
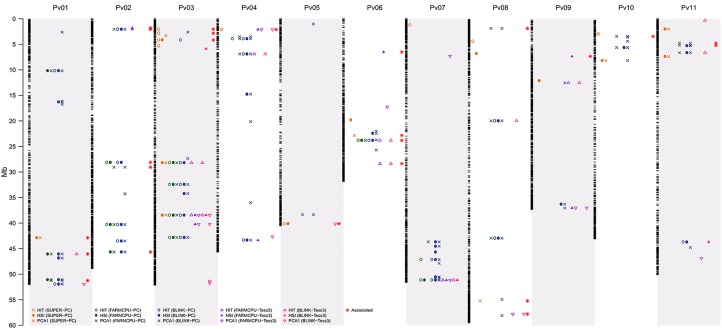
Physical map of the 23,373 SNP markers for all 11 common bean (Pv) chromosomes. Physical position is shown in millions of base pairs (Mb). Each black hyphen corresponds to a SNP marker. The columns of yellow plotting symbols indicate markers associated by the SUPER algorithm with HIT (circle), HSI (filled circle), and PCA1 (cross) indices, using EMMA as a random effect and PC as a fixed effect (**Figures 2E, F**, **3E, F**, and **4E, F**). The columns of green plotting symbols indicate markers associated by the FarmCPU algorithm with HIT (circle), HSI (filled circle), and PCA1 (cross) indices, using EMMA as a random effect and PC as a fixed effect (**Figures 2G, H**, **3G, H**, and **4G, H**). The columns of blue plotting symbols indicate markers associated by the BLINK algorithm with HIT (circle), HSI (filled circle), and PCA1 (cross) indices, using EMMA as a random effect and PC as a fixed effect (**Figures 2I, J**, **3I, J**, and **4I, J**). The columns of purple plotting symbols indicate markers associated by the FarmCPU algorithm with HIT (triangle point up), HSI (filled triangle point-up blue), and PCA1 (triangle point down) indices, using EMMA as a random effect and TESS3 as a fixed effect (**Figure 5A, F**). The columns of pink plotting symbols indicate markers associated by BLINK algorithm with HIT (triangle point up), HSI (filled triangle point-up blue), and PCA1 (triangle point down) indices, using EMMA as a random effect and TESS3 as a fixed effect (**Figure 5G,L**). The columns of red stars indicate markers associated with genes related with the HS response, such as activation of heat shock proteins (HSPs) and abiotic stress signaling. Regions are defined here as overlapping 1,000-bp sections that flanked associated markers (**Table 2**).

We implemented a GLM model in the first step of the SUPER algorithm as suggested by Wang et al. (2014b) due to its computational efficiency, same as MLM and CMLM models. MLM and CMLM models were implemented for the last step of the SUPER algorithm with each of the three HS indices. From all these 12 SUPER models, the only ones that reported associated markers at a Bonferroni threshold of −log_10_ *P*-value = 5.67 were HSI_SUPER(CMLM)-PC-EMMA_ (**Figures 2E, F**), HIT_SUPER(CMLM)-PC-EMMA_ (**Figures 3E, F**), and PCA1_SUPER(CMLM)-PC-EMMA_ (**Figures 4E, F**), from now on named as HIT_SUPER-PC-EMMA_, HSI_SUPER-PC-EMMA_, and PCA1_SUPER-PC-EMMA_, respectively, for better reading. The remaining nine SUPER models [HIT_SUPER(CMLM)-TESS3-EMMA_, HSI_SUPER(CMLM)-TESS3-EMMA_, PCA1_SUPER(CMLM)-TESS3-EMMA_, HIT_SUPER(MLM)-PC-EMMA_, HSI_SUPER(MLM)-PC-EMMA_, PCA1_SUPER(MLM)-PC-EMMA_, HIT_SUPER(MLM)-TESS3-EMMA_, HSI_SUPER(MLM)-TESS3-EMMA_, and PCA1_SUPER(MLM)-TESS3-EMMA_], abbreviated as “failed” SUPER models, only identified between 17 and 12 SNP markers that crossed the lax threshold of −log_10_ *P*-value = 4 (**Figures S7A–P**). On the other hand, all 12 FarmCPU (**Figures 2**–**4G, H** and **5A–F**) and BLINK (**Figures 2**–**4I, J** and **5G–L**) models reported associated markers at a Bonferroni threshold of −log_10_ *P*-value = 5.23. Regardless what population stratification method was used (PC or TESS3) as a fixed effect, the 15 last-generation models SUPER (three), FarmCPU (six), and BLINK (six) identified a total of 120 associated loci at a Bonferroni threshold (**Table 1**). A total of 61 from the 120 SNP markers were captured by a single GEA model, and the remaining 59 SNP markers were associated with more than one of these GEA models; thus, we obtained a total of 270 GWAS redundant outputs (**Table S3**). The 120 significantly associated SNP markers were distributed in 105 regions across the common bean genome (**Figure 6**). Chromosomes Pv03, Pv01, Pv11, and Pv07 harbored the highest number of markers with 18, 15, 14, and 14 SNPs in 16, 12, 11, and 12 regions, respectively. Chromosomes Pv06, Pv08, Pv04, Pv02, and Pv10 had 10, 10, 10, 9, and 9 associated markers grouped in 10, 9, 10, 9, and 6 regions, respectively. Pv09 and Pv05 were the chromosomes with the fewest associated markers with seven and four SNPs, grouped in six and four regions (**Table S4**). On the other hand, PCA1 was the HS index with the highest number of markers with 96 SNPs in 83 regions through the entire genome. The HS indices HSI and HIT had 57 and 37 associated markers grouped in 54 and 33 regions, respectively, across all chromosomes (**Table S4**). Also, the last-generation GWAS algorithm with the highest number of associated markers was BLINK with 91 SNPs in 80 regions through the entire genome. The FarmCPU and SUPER algorithms had 46 and 24 associated markers grouped in 44 and 21 regions, respectively, across all chromosomes (**Table S4**).

**Table 1:** Summary statistics of the 15 gene–environment association (GEA) models for the 120 single-nucleotide polymorphism (SNP) markers associated with the three heat stress (HS) indices (HSI, HIT, and PCA1) in 78 common bean accessions based on the optimum association analysis (**Figures 2**–**5**).

Model	Pv	Number of associated SNPs	Average −log_10_ (*P*-value)	Average *R* ^2^ (%)	Number of exclusive associated SNPs	Number of associated regions	Number of associated regions containing genes	Number of genes	Number of genes related to HS
HIT_BLINK-PC-EMMA_	1, 2, 3, 4, 6, 7, 8, 11	21	8.0 ± 1.1	64.62 ± 0.10	0	20	6	6	3
HIT_BLINK-TESS3-EMMA_	1, 3, 4, 6, 7, 8, 9, 11	12	7.9 ± 0.8	60.86 ± 0.07	1	11	3	3	3
HIT_FARMCPU-PC-EMMA_	2, 3, 4, 6, 7	11	6.8 ± 0.5	68.71 ± 0.10	0	10	4	4	2
HIT_FARMCPU-TESS3-EMMA_	3, 4, 6, 7, 9	8	7.5 ± 0.2	57.09 ± 0.09	0	7	2	2	2
HIT_SUPER(CMLM)-PC-EMMA_	3, 4, 5, 7, 8, 10	12	7.0 ± 0.3	26.71 ± 0.02	9	9	5	5	5
HSI_BLINK-PC-EMMA_	1, 2, 3, 4, 6, 7, 8, 9, 10, 11	39	8.2 ± 0.8	59.91 ± 0.10	1	36	9	9	4
HSI_BLINK-TESS3-EMMA_	3, 7, 11	5	7.3 ± 0.6	63.36 ± 0.06	1	4	0	0	0
HSI_FARMCPU-PC-EMMA_	1, 2, 3, 6, 7	12	6.5 ± 0.5	67.83 ± 0.10	0	12	6	6	4
HSI_FARMCPU-TESS3-EMMA_	2, 3, 4, 6, 7, 9	10	7.4 ± 0.9	52.58 ± 0.18	5	10	5	5	5
HSI_SUPER(CMLM)-PC-EMMA_	1, 3, 5, 6, 8, 9, 10, 11	11	6.8 ± 0.6	26.10 ± 0.05	3	11	4	4	3
PCA1_BLINK-PC-EMMA_	1, 2, 3, 4, 5, 6, 7, 8, 9, 10, 11	73	7.8 ± 0.8	56.44 ± 0.08	30	62	16	18	8
PCA1_BLINK-TESS3-EMMA_	1, 3, 4, 5, 8, 9	11	7.0 ± 0.7	48.59 ± 0.16	5	10	5	5	2
PCA1_FARMCPU-PC-EMMA_	1, 2, 3, 4, 5, 6, 7, 8, 10, 11	27	6.6 ± 0.4	61.19 ± 0.09	0	26	14	14	9
PCA1_FARMCPU-TESS3-EMMA_	3, 4, 6, 7, 8, 9, 11	9	6.6 ± 0.9	47.82 ± 0.13	3	9	2	2	2
PCA1_SUPER(CMLM)-PC-EMMA_	1, 3, 6, 8, 10, 11	9	6.8 ± 0.6	42.86 ± 0.02	3	9	2	4	3

From 15 significant-GEA models, PCA1_BLINK-PC-EMMA_, HSI_BLINK-PC-EMMA_, PCA1_FARMCPU-PC-EMMA_, and HIT_BLINK-PC-EMMA_ were the models with the highest number of markers with 73, 39, 27, and 21 SNPs in 62, 36, 26, and 20 regions, respectively, through the entire genome. The models HIT_BLINK-TESS3-EMMA_, HIT_SUPER(CMLM)-PC-EMMA_, HSI_FARMCPU-PC-EMMA_, HIT_FARMCPU-PC-EMMA_, HSI_SUPER(CMLM)-PC-EMMA_, PCA1_BLINK-TESS3-EMMA_, and HSI_FARMCPU-TESS3-EMMA_ had 12, 12, 12, 11, 11, 11, and 10 SNPs in 11, 9, 12, 10, 11, 10, and 10 regions, respectively, across all chromosomes. PCA1_FARMCPU-TESS3-EMMA_, PCA1_SUPER(CMLM)-PC-EMMA_, HIT_FARMCPU-TESS3-EMMA_, and HSI_BLINK-TESS3-EMMA_ were the models with the fewest associated markers with nine, nine, eight, and five SNPs, grouped in nine, nine, seven, and four regions, respectively (**Table 1**).

Also, the models PCA1_BLINK-PC-EMMA_, HIT_SUPER(CMLM)-PC-EMMA_, PCA1_BLINK-TESS3-EMMA_, and HSI_FARMCPU-TESS3-EMMA_ had the highest number of exclusive markers that no other model captured, with 30, 9, 5, and 5 SNPs, respectively. The models HSI_SUPER(CMLM)-PC-EMMA_, PCA1_FARMCPU-TESS3-EMMA_, PCA1_SUPER(CMLM)-PC-EMMA_, HSI_BLINK-PC-EMMA_, HIT_BLINK-TESS3-EMMA_, and HSI_BLINK-TESS3-EMMA_ had the fewest exclusive markers with three, three, three, one, one, and one SNPs, respectively. The remaining models from the 15 significant-GEA models did not have exclusive SNP markers (**Table 1**). On the other hand, the 120 significantly associated SNP markers explained 54.28%, 52.73%, and 51.73% of the variation (effects) for PCA1, his, and HIT, respectively (**Table S4**). Furthermore, we averaged the *R* ^2^ of all associated SNPs by each of the significant 15 models throughout the genome of common bean, getting a range of average effects between 68.71% (HIT_FARMCPU-PC-EMMA_) and 26.10% (HSI_SUPER(CMLM)-PC-EMMA_) (**Table 1**). In summary, from the entire set of 30 GEA models implemented with improved traditional MLMs and last-generation GWAS algorithms, only 15 reported associations at a Bonferroni threshold, for a total of 120 associated markers.

### Associated Markers Flanked 22 Genes Related With the HS Response, Such as Activation of HSPs and Abiotic Stress Signaling

We identified 120 associated loci across 15 of the 30 run GEA models at a Bonferroni-corrected significance threshold of −log10 P-value = 5.23 for 12 FarmCPU and BLINK models and at a Bonferroni-corrected threshold of −log10 P-value = 5.67 for three SUPER models. Among the 15 GEA models that captured significantly associated markers, only one (HITBLINK-TESS3-EMMA) did not identify any flanking gene. The other 14 models captured 36 flanking genes (**Table S3**). An ontology analysis revealed that 22 of these genes, flanking 24 associated markers, related with biological processes of the response to heat tolerance in plants (**Figure 6**, **Table 2**).

**Table 2:** List of 24 single-nucleotide polymorphism (SNP) markers associated and flanked (genomic window of 1 kb) to 22 genes related with the heat stress (HS) response such as activation of heat shock proteins (22.73%), abiotic stress signaling (18.18%), germination and seedling development (18.18%), flowering time (9.09%), protein domain thermostability (9.09%), molecular chaperones (9.09%), and stability of the cell wall (4.55%) using PhytoMine B and reference genome of common bean v2.1.

Gene Name	GEA Model	Associated SNPs	Gen
**Activation of heat shock proteins—five genes (22.73%)**			
Phvul.003G021100	HIT_SUPER(CMLM)-PC-EMMA_	S1_103273611	***MED23***
Phvul.003G028300	HIT_SUPER(CMLM)-PC-EMMA_	S1_104075622	***MED25***
Phvul.003G038600	HIT_SUPER(CMLM)-PC-EMMA,_ HSI_SUPER(CMLM)-PC-EMMA_, HIT_BLINK-PC-EMMA_	S1_105404421	***Hsp40*** *—*Pv03
Phvul.002G136100	HSI_FARMCPU-PC-EMMA_, HIT_FARMCPU-PC-EMMA_, HSI_BLINK-PC-EMMA_, HIT _BLINK-PC-EMMA_	S1_80309359	***Hsp40***—Pv02
Phvul.006G182100	HIT_FARMCPU-TESS3-EMMA_, HIT_BLINK-TESS3-EMMA_	S1_268677251	***Hsp40***—Pv06
Phvul.002G019100	HSI_FARMCPU-TESS3-EMMA_	S1_54254560	***HSFB1*** *(HSF4)*
Phvul.008G227900	PCA1_FARMCPU-TESS3-EMMA_, PCA1_BLINK-TESS3-EMMA_	S1_381855152	***HSP20***
**Abiotic stress signaling—four genes (18.18%)**			
Phvul.008G204500	PCA1_SUPER(CMLM)-PC-EMMA_	S1_379270378	***NFY***
Phvul.002G142500	PCA1_FARMCPU-PC-EMMA_, PCA1_BLINK-PC-EMMA_	S1_81263655	***AUX_IAA***
Phvul.006G014100	HSI_FARMCPU-TESS3-EMMA_	S1_246823134	Phospholipase C***PLC***
Phvul.001G202000	HSI_FARMCPU-PC-EMMA_, PCA1_FARMCPU-PC-EMMA_, HSI_BLINK-PC-EMMA_, PCA1_BLINK-PC-EMMA_, HIT_BLINK-TESS3-EMMA_	S1_46052073	Auxin response factor
**Germination and seedling development–four genes (18.18%)**			
Phvul.005G175800	HSI_SUPER(CMLM)-PC-EMMA_, HIT_SUPER(CMLM)-PC-EMMA_	S1_239620265	Glycoside hydrolases family 28
Phvul.002G016700	HSI_FARMCPU-TESS3-EMMA_	S1_53999562	Family ***AP2/ERF***
Phvul.001G171600	HSI_SUPER(CMLM)-PC-EMMA_, PCA1_SUPER(CMLM)-PC-EMMA_	S1_42870591	***Ankyrin-B (Ankyrin-2)***
Phvul.011G054400	PCA1_FARMCPU-PC-EMMA_, PCA1_BLINK-PC-EMMA_	S1_469234219	***Pkinase_Tyr***
**Flowering time—two genes (9.09%)**			
Phvul.006G119900	PCA1_SUPER(CMLM)-PC-EMMA_	S1_263134744	Poly(A) polymerase ***PAP***
Phvul.004G017600	HIT_SUPER(CMLM)-PC-EMMA_, PCA1_FARMCPU-TESS3-EMMA_, HSI_FARMCPU-TESS3-EMMA_, PCA1_BLINK-TESS3-EMMA_	S1_155643598	***MBD9***
**Protein domain thermostability—two genes (9.09%)**			
Phvul.011G058100	PCA1_FARMCPU-PC-EMMA_, PCA1_BLINK-PC-EMMA_	S1_469639214	Zinc finger A20 and AN1
Phvul.002G287600	PCA1_FARMCPU-PC-EMMA_, HSI_FARMCPU-PC-EMMA_, HSI_BLINK-PC-EMMA_, PCA1_BLINK-PC-EMMA_	S1_97861798	S1
**Molecular chaperones—two genes (9. 09%)**			
Phvul.009G032500	HSI_FARMCPU-TESS3-EMMA_	S1_391075082	***14-3-3*** proteins family
Phvul.010G024400	PCA1_FARMCPU-PC-EMMA_, PCA1_BLINK-PC-EMMA_	S1_424636676	***FKBP***
**DNA transcription—two genes (9. 09%)**			
Phvul.008G022950	PCA1_FARMCPU-PC-EMMA_, PCA1_BLINK-PC-EMMA_	S1_325947871	***BRIX***
Phvul.006G130200	HSI_FARMCPU-PC-EMMA_, HIT_FARMCPU-PC-EMMA_, PCA1_FARMCPU-PC-EMMA_, HSI_BLINK-PC-EMMA_, HIT_BLINK-PC-EMMA_, HIT_FARMCPU-TESS3-EMMA_, HIT_BLINK-TESS3-EMMA_	S1_264118438	***YTH*** protein domain
**Stability of the cell wall—one gene (4.55%)**			
Phvul.001G267000	PCA1_FARMCPU-PC-EMMA_, PCA1_BLINK-PC-EMMA_	S1_51236796	***GAE6***

B https://phytozome.jgi.doe.gov/pz/portal.html

The chromosomes with the highest number of genes related to heat tolerance were Pv02, Pv06, Pv03, Pv01, Pv08, and Pv11, with five, four, three, three, three, and two genes, respectively (**Table S4**). The chromosomes with only one gene related to heat tolerance were Pv04, Pv05, Pv09, and Pv10. Pv07 was the only chromosome that did not report gene related to heat tolerance. On the other hand, the PCA1 was the HS index with the highest number of genes related to heat tolerance with 14 genes. Furthermore, the HS indices HSI and HIT had 12 and 9 genes related to heat tolerance, respectively (**Table S4**). Also, the last-generation GWAS algorithm with the highest number of genes related to heat tolerance was FarmCPU with 18 genes. The BLINK and SUPER algorithms had 14 and 8 genes related to heat tolerance, respectively (**Table S4**).

From 15 significant-GEA models, PCA1_FARMCPU-PC-EMMA_, PCA1_BLINK-PC-EMMA_, HIT_SUPER(CMLM)-PC-EMMA_, HSI_FARMCPU-TESS3-EMMA_, HSI_BLINK-PC-EMMA_, and HSI_FARMCPU-PC-EMMA_ were the models with the highest number of genes related to heat tolerance with nine, eight, five, five, four, and four genes, respectively. On the other hand, HSI_SUPER(CMLM)-PC-EMMA_, PCA1_SUPER(CMLM)-PC-EMMA_, HIT_BLINK-TESS3-EMMA_, HIT_BLINK-PC-EMMA_, PCA1_FARMCPU-TESS3-EMMA_, PCA1_BLINK-TESS3-EMMA_, HIT_FARMCPU-TESS3-EMMA_, and HIT_FARMCPU-PC-EMMA_ were the models with the fewest number of genes related to heat tolerance with three, three, three, three, two, two, two, and two genes, respectively. HSI_BLINK-TESS3-EMMA_ was the only GEA model that had no associated genes (**Table 1**).

A total of 22 genes flanked 24 loci because three different copies of the *HSP40* (Wang et al., 2004) gene were reported on three different chromosomes (Pv02, Pv03, and Pv06) using eight GEA models that incorporated HIT and HSI as response environmental variables. Four other genes from the set of 22 were also related to pathways of response to HS, such as activation of HSPs [*MED23* (Kim et al., 2004), *MED25* (Mathur et al., 2011), and *HSFB1* (Ikeda et al., 2011) in Pv02; and *HSP20* (Lopes-Caitar et al., 2013) in Pv08]. This set of five genes (*HSP20*, *HSP40*, *MED23*, *MED25*, and *HSFB1*) was recovered by 11 redundant GEA models [HIT_BLINK-PC-EMMA_, HIT_BLINK-TESS3-EMMA_, HIT_FARMCPU-PC-EMMA_, HIT_FARMCPU-TESS3-EMMA_, HIT_SUPER(CMLM)-PC-EMMA_, HSI_SUPER(CMLM)-PC-EMMA_, HSI_BLINK-PC-EMMA_, HSI_FARMCPU-PC-EMMA_, HSI_FARMCPU-TESS3-EMMA_, PCA1_BLINK-TESS3-EMMA_, and PCA1_FARMCPU-TESS3-EMMA_] (**Table 2**). These precursor genes of HSPs can play a crucial role in protecting plants against stress by reestablishing normal protein conformation and thus cellular homeostasis (Wang et al., 2004). Four significant SNP markers were found within the coding sequencing of the duplicated *HSP40* genes (Pv02 and Pv06), *MED23* and *MED25*. We also found two genes associated with protein domains related to thermostability in plants such as *S1* in Pv02 and *Zinc finger A20 and AN1* (Dixit and Dhankher, 2011) in Pv11 (**Table 2**).

We also recovered nine genes associated with biological processes likely correlated with plant tolerance to high temperatures, such as flowering time (*MBD9* in Pv04 and *PAP* in Pv06) (Peng et al., 2006; Trost et al., 2014), regulation of molecular chaperones (*FKBP* in PV10 and *14-3-3* proteins in Pv09) (Wang et al., 2004; Gollan et al., 2012), germination and seedling development [*Pkinase_Tyr* family in Pv11, *Ankyrin-B* in Pv01 (Hanks and Quinn, 1991; Bae et al., 2008), glycoside hydrolase *GH* family (González-Carranza et al., 2002) in Pv05, and transcription factors family *AP2/ERF* (Jofuku et al., 1994; Büttner and Singh, 1997) in Pv02], and cell wall stability (*GAE6* in Pv01) (Usadel et al., 2004). Additionally, four genes were involved in the signaling pathways of abiotic stress via abscisic acid [histone-like transcription factors *NFYB* (Warpeha et al., 2007) in Pv08 and phospholipase C *PLC* (Peters et al., 2010) in Pv06] and auxin (auxin response factor in Pv01 and *AUX_IAA* in Pv02) (Hagen and Guilfoyle, 2002; Ellis et al., 2005) (**Table 2**). On the other hand, since HS compromises molecular processes inherent to DNA transcription, it is not unexpected that we found two transcription factors [*BRIX* (Weis et al., 2015) in Pv08 and protein domains *YTH* (Wang et al., 2014a) in Pv06] involved in plant development and response to abiotic stress such as drought and heat. Overall, the biological processes related to HS over-represented among the associated genes were thermal shock protein activation (22.73%), abiotic stress signaling (18.18%), and germination and seedling development (18.18%) (**Table 2**).

Additionally, we explored a genomic window of 81 kb (40.5 kb upstream to 40.5 kb downstream of the associated SNP using the common bean v2.1, **Table S5**) based on LD criterion, finding 541 new genes for a total of 578 genes. Among the 578 genes, we found eight genes related to HSPs (three *HSP40*, two *HSP20*, one *HSFA5*, and two *HSP17.6*) in addition to the five genes found in the window of 1 kb (three *HSP40*, one *HSP20*, one *HSFB1*, one *MED23*, and one *MED25*) for a total of thirteen genes. The eight new genes related to HSP were distributed like this: three *HSP40* in chromosomes Pv01, Pv06, and Pv07; two *HSP20* in chromosomes Pv05 and Pv08; one *HSFA5* in chromosome in Pv01; and two *HSP17.6* in chromosome Pv08.

### Last-Generation GWAS Models Complemented Each Other Despite Some Redundancy

Based on the previous gene recovery and classification, 11 GEA models were the best at explaining the activation of HSPs as the genetic basis of heat tolerance, by reporting seven loci across five chromosomes (**Tables 2** and **S3**) as potential candidates to be integrated into breeding programs. These seven loci were related to genes belonging to the HSPs’ activation signaling pathway. From these 11 GEA models, the ones that best explained the HS indices were HIT_FARMCPU-PC-EMMA_ (68.71%), HSI_FARMCPU-PC-EMMA_ (67.83%), and PCA1_FARMCPU-PC-EMMA_ (61.19%). In other words, the last-generation GWAS model families that best explained the HS indices were FarmCPU and BLINK. Meanwhile, SUPER models reported the weakest effects (42.86%) (**Table 1**).

Among the 11 most-explanatory GEA models, 10 models, distributed in four main clusters, were redundant. HSI_FARMCPU-TESS3-EMMA_ was the unique non-redundant model that captured a gene related to heat tolerance (*HSFB1*) (**Table 2**). The clustering criterion was that models within the same cluster captured the same gene. The first cluster had three models (HIT_SUPER-PC-EMMA_, HSI_SUPER-PC-EMMA_, and HIT_BLINK-PC-EMMA_) that reported a paralogous copy of the *HSP40* gene in chromosome Pv03. The second cluster had four models (HIT_BLINK-PC-EMMA_, HSI_FARMCPU-PC-EMMA_, HIT_FARMCPU-PC-EMMA_, and HSI_BLINK-PC-EMMA_) that reported a paralogous of the same gene in chromosome Pv02. The third cluster had two models (HIT_FARMCPU-TESS3-EMMA_ and HIT_BLINK-TESS3-EMMA_) that identified the other paralogues of *HSP40* in chromosome Pv06. The fourth cluster was made of two models (PCA1_FARMCPU-TESS3-EMMA_ and PCA1_BLINK-TESS3-EMMA_) that captured the same *HSP20* gene in chromosome Pv08. On the other hand, the genes that were captured by non-redundant models were *MED23* and *MED25* (both with HIT_SUPER-PC-EMMA_) and *HSFB1* (with HSI_FARMCPU-TESS3-EMMA_). The HIT_SUPER-PC-EMMA_ model was not redundant with other models when capturing these two genes, but this model was redundant with the first cluster when capturing the Pv02 *HSP40* paralogues.

The HIT_BLINK-PC-EMMA_ model simultaneously reported the paralogous *HSP40* gene in chromosomes Pv02 (SNP marker S1_80309359, effect = 56.22%) and Pv03 (SNP marker S1_105404421, effect = 56.74%), from the first and second clusters, respectively. The LD between both SNP markers reported by HIT_BLINK-PC-EMMA_ had an *R* ^2^ of 6.2% (*P*-value = 0.045). In other words, both SNP markers were recovered by the same model (HIT_BLINK-PC-EMMA_) and accounted for different effects of paralogous copies of the *HSP40* gene in different chromosomes. So we selected the HIT_BLINK-PC-EMMA_ model as the representative model of the first and second clusters. On the other hand, we selected the most explanatory models (highest effects) as representative models for the third and fourth clusters. Thus, we chose the HIT_BLINK-TESS3-EMMA_ model (effect = 60.86%) as the representative model of the third cluster (**Table 1**). This model identified the *HSP40* gene in chromosome Pv06. Finally, we selected the PCA1_BLINK-TESS3-EMMA_ model (effect = 48.59%) as the representative model of the fourth cluster (**Table 1**). This model captured the *HSP20* gene in chromosome Pv08. Therefore, the 11 models that best explained the activation of HSPs can be condensed into five non-redundant models, which are HIT_BLINK-PC-EMMA_, HIT_SUPER-PC-EMMA_, HSI_FARMCPU-TESS3-EMMA_, HIT_BLINK-TESS3-EMMA_, and PCA1_BLINK-TESS3-EMMA_. Each of these five non-redundant GEA models captured a unique gene of the HSPs’ activation signaling pathway, including regulators of mediators, activators, and expression genes (HIT_BLINK-PC-EMMA_ captured *HSP40* in Pv03 and Pv02, HIT_SUPER-PC-EMMA_ captured *MED23* and *MED25*, HSI_FARMCPU-TESS3-EMMA_ captured *HSFB1*, HIT_BLINK-TESS3-EMMA_ captured *HSP40* in Pv06, and PCA1_BLINK-TESS3-EMMA_ captured *HSP20*) (**Table 2**).

## Discussion

The discriminatory power provided by kinship covariates used as random effects has been of great interest in the development of promising GWAS algorithms (CMLM, SUPER, FarmCPU, and BLINK). However, last-generation GWAS algorithms have given greater importance to the selection of SNP markers for the kinship reconstruction than to the reconstruction method itself. The three HS indices (HIT, HSI, and PCA1) and 15 last-generation GWAS models that generated significant results captured complementary components of the genetic architecture of heat tolerance. We found a total of 24 loci associated to 22 genes related to biological processes of the HS response in plants. Also, among the 24 loci, we captured seven loci as potential candidates to be integrated into breeding programs, since they were flanking five genes belonging to the signaling pathway that activates HSPs.

### Bioclimatic Indices Capture Complementary Genetic Effects Conferring Heat Tolerance

Each HS index captures a different facet of the HS event. The HIT index uses accumulated information of maximum temperatures during the reproductive phase of common bean and therefore is more informative over time in capturing extreme values related to HS. Because of this dynamic nature of HIT, models that integrated HIT were more successful at capturing genes related to the activation of HSPs. In addition, the HIT_SUPER-PC-EMMA_ model, which integrates the HIT index as a response variable, captured unique results that no other model recovered, by reporting key genes in the activation of HSPs such as *MED23* and *MED25* activators, which are key genes in the reconstruction of the genetic basis of heat tolerance.

On the other hand, the HIS index is built on thresholds of maximum temperature during the reproductive phase reported by some authors for plants in the tropics (Gonçalves et al., 1997; Caramori et al., 2001; Silva et al., 2007; Rainey and Griffiths, 2019). Thus, this index could be more informative phenologically in capturing extreme values related to HS events. This is based on the fact that models constructed with HIS tended to capture more unique genes than any other index. Furthermore, HSI_FARMCPU-TESS3-EMMA_ was the only model that captured the HS gene heat shock factor *HSFB1* (*HSF4*). Among the set of genes captured by 11 GEA models, *HSF4*, a regulatory gene in the expression of HSPs in *Arabidopsis thaliana* (Ikeda et al., 2011), is the gene that has greater regulatory importance both in the activation of HSPs and other molecular mechanisms of response to abiotic stress. Then, although the HIS index fails to capture the amount of genes that the HIT index did, perhaps because of its stationary nature, it manages to identify unique results that are essential to reconstruct the complexity of the genetic effects that confer heat tolerance.

Finally, the index based on PCA1 exhibits variability that the first two indices did not offer. PCA1 integrates other bioclimatic variables besides *T* *_j_*, yet still related to abiotic stress events. The wide variability offered by PCA1 is evident in the large coverage of the GEA models that relied on this index. These models capture more candidate genes than the previous ones (14 from 22 genes). They also capture more biological processes related to HS (*e.g.* abiotic stress signaling, germination and development of seedlings and flowering time). However, they recover few genes related with the activation of HSPs proteins. The models PCA1_FARMCPU-TESS3-EMMA_ and PCA1_BLINK-TESS3-EMMA_ capture unique genes such as *HSP20*, reported in soybean as activator of HSPs (Lopes-Caitar et al., 2013), and reported in common bean as one of the three most over-expressed genes under HS using RNA-sequencing (Soltani et al., 2019).

Each index captures unique genes associated with the activation of HSPs, but each of them also identifies different paralogous copies of the same gene. The models that used the HSI and HIT indices, recover genes *upstream* to HSPs genes in the pathway of activation of HSPs (*i.e. HSFB1*, *MED23*, and *MED25*). On the other hand, genes of the family of low molecular weight sHSPs (small HSPs), such as *HSP40*, are found in Pv02 and Pv03 chromosomes using the HIT and HSI indices, and in Pv06 chromosome using the HIT. Also, other low molecular weight HSPs such as *HSP20* are captured by the PCA1 index. Thus, models that integrate different indices manage to identify mediating, activating and expression genes (sHSPs), providing a more comprehensive understanding of the genetic architecture of heat tolerance. Although the three indices fail to capture all the conserved families of HSPs such as *Hsp70*, *Hsp60*, *Hsp90* and *Hsp100*, they detect associations flanking several genes of the family sHSPs, such as *HSP20* and *HSP40*, this is possibly because sHSP family is the most prevalent in plants (Vierling, 1991). In addition, gene diversification and subspecialization may reflect molecular adaptation to stress conditions that are unique to specific populations (Wang et al., 2004). On the other hand, high abundance of sHSPs in multiple cellular compartments suggests that they may have an important role in acquisition of stress tolerance in plants (Wang et al., 2004). In this sense, the expression of sHSPs genes, as those detected in this study by means of the three different indices, despite not being the proteins that have higher folding potential (as *Hsp70* and *Hsp90)*, can be key regulatory steps of the molecular response to HS (by modulating genes such as *HSFB1*, *MED23*, and *MED25*, that we also managed to detect).

### An Assortment of Various Last-Generation GWAS Models Offer Better Alternatives for GEA Studies

Each last-generation GWAS algorithm implemented in this study differs in the internal strategy that uses to reconstruct the random Kinship covariable. While the kinship method is consistent across algorithms, the implementation of Pseudo QTNs differs. On the other hand, a prerequisite for GWAS models is the use of fixed covariables for population structure, being the principal components analysis (PC) the most traditional method. However, the generation of alternative strategies such as the one implemented in TESS3, which is more powerful to reconstruct the stratification of the population as evidenced by the works of Caye et al. (2016), Ariani et al. (2018) and Varshney et al. (2017), led us to consider TESS3 as a promising method to be integrated into the GEA models. The results obtained by models that use TESS3 as a fixed covariate, demonstrate its importance to capture candidate genes not recovered by any other GEA model, such as an activator of HSP proteins (*HSFB1*) and two HSPs of low molecular weight (*HSP20*, and *HSP40* in Pv06). On the other hand, the implementation of the PC method as a fixed covariate in GEA models is also useful, because the models that integrate this method capture unique genes such as HSPs of low molecular weight (*HSP40* genes in Pv02 and Pv03) and activators of HSP proteins (*MED23* and *MED25*).

In summary, 30 GEA models were built with TESS3 and PC as fixed covariables, from an improved traditional MLM algorithm (CMLM) and three last-generation GWAS models (SUPER, FarmCPU, and BLINK). Of the 30 GEA models, 14 used last GWAS algorithms and reported genes linked to biological processes related to HS. A total of 11 of these 14 models captured genes related to molecular mechanism of activation of HSPs proteins. This molecular process was given greater focus due to its importance for heat tolerance and its relationship with other stresses. The 11 GEA models that identified HSP activation genes can be condensed into five non-redundant GEA models, conserving the same number of associated genes.

We did not find the ‘holy grail’ for GWAS models, which is a unique model that would summarize all 14 GEA models. The majority of the 14 GEA models that used FarmCPU and BLINK algorithms are redundant in the results related to activation of HSPs, regardless whether these considered TESS3 or PC as fixed covariables. The coincidence between the results obtained by FarmCPU and BLINK had already been reported by the authors of the BLINK algorithm for flowering time in corn (Huang et al., 2019), and was attributed to the way both strategies are conceived. They operate by separating the mixed model into a fixed sub-model and a random sub-model, differing only in the parameter-estimation method (Huang et al., 2019). This is why both methodologies converge to the same results for heat tolerance in common beans and flowering time in corn. However, in our study an exception to the redundancy between HIT_BLINK-PC-EMMA_ and HIT_FARMCPU-PC-EMMA_ algorithms was that the exact identity of the associated markers within the candidate genes differed. Besides, despite that the authors of BLINK reported that this method captures more associated genes to flowering time than FarmCPU, we found the opposite pattern when it comes to heat tolerance in common bean. This suggests that the algorithms could be sensible to the use of different response variables (*e.g.* environmental vs. phenotypic).

The Q–Q plot can provide information on two main aspects of GWAS data: whether the statistical testing is well controlled for challenges such as population stratification and whether there is any association. In the last aspect, we could see some associations at the end of the Q–Q plot crossing the Bonferroni threshold. The population structure control is still a challenge in GWAS and our Q–Q plots show signs of inflation. This inflation could partially be produced by causal SNPs (or SNPs in LD with causal variants), that at the same time are strongly differentiated among gene pools. This scenario is possible because both gene pools come from contrasting environments in terms of exposure to HS events. Mesoamerican genotypes generally experience more heat events than Andean genotypes (**Figure S8**).

In conclusion, the five non-redundant GEA models that best explain the activation of HSPs as the genetic basis of heat tolerance are HIT_BLINK-PC-EMMA_, HIT_SUPER-PC-EMMA_, HSI_FARMCPU-TESS3-EMMA_, HIT_BLINK-TESS3-EMMA_ and PCA1_BLINK-TESS3-EMMA_. Each of these models captures a key gene in the pathway of activation of sHSPs, including genes involved in the regulation, activation and expression of the signal (Vierling, 1991). Therefore, using an assortment of last-generation GWAS methods, various environmental indices and different methods to account for fixed covariates, is much more informative than trying to select a single optimum GWAS model. Our work presents for the first time a powerful strategy to explore GEAs throughout a wide range of different last-generation GWAS models. This opens the door for new ways to couple environmental information in the study of complex characters, such as heat tolerance.

### Modern GEA Is Capable of Revealing the Genetic Basis of a Complex Adaptive Trait Despite Limited Sampling

HS affects several physiological, cellular and molecular processes in plant cells, affecting fluidity of the cell membrane (Savchenko et al., 2002), protein (Ahmad et al., 2009) and cytoskeletal stability (Bita and Gerats, 2013), chromatin structure (Khraiwesh et al., 2012), the production of reactive oxygen species (ROS) (Camejo et al., 2006) as well as metabolic coupling (Bita and Gerats, 2013). Consequently, HS generates responses in plant cells at molecular and cellular levels, such as activation of HSPs (Wang et al., 2004), calcium signaling (Larkindale and Huang, 2004), phosphorylation, changes in the transcription (Bita and Gerats, 2013) and hormonal responses via Abscisic Acid (Larkindale and Knight, 2002), Ethylene or Auxin (Evrard et al., 2013; Larkindale and Huang, 2004). Yet, HS also affects processes such as flowering time, germination and abscission of floral organs (Bita and Gerats, 2013). The genes reported in this work may be causal or in LD with causal genes, involved in the majority of these processes. Although, we captured at least one gene in each of these biological processes, the highest number of associated genes were involved in the activation of HSPs. This could be attributed to the ability of the sHSPs family (*e.g. HSP20* and *HSP40*) and HSF genes (*HSFB1*) to activate HSPs as well as other physiological, cellular, and molecular mechanisms of heat tolerance in plants, such as hormonal signaling routes (Wang et al., 2004), photosystem II protection (Kotak et al., 2007, Soltani et al., 2019), DNA translation control (Malik et al., 1999) and elimination of reactive oxygen species (ROS) (Bita and Gerats, 2013). In addition, if we focused in genes related to HSPs, the resolution to detect these proteins decreases with a wider window of 81 kb. Among the 578 genes, we found eight genes related to HSPs (three HSP40, two HSP20, one HSFA5, and two HSP17.6) in addition to the five genes found in the windows of 1 kb (three HSP40, one HSP20, one HSFB1, one MED23, and one MED25) for a total of thirteen genes. However, these thirteen genes are the 2,25 % of the 578 genes found in a genomic window of 81 kb, while in a narrower genomic window of 1 kb, the five genes related to HSP are the 13.5% of the 37 genes.

Although we were unable to reconstruct the entire pathways of HSP protein activation, hormonal responses, time to flowering and seedling development, we found key genes in these biological processes, by only using environmental information from the accession’s sampling sites. This strategy is valuable in optimizing time and costs for association studies using wild material.

We have demonstrated that combining diverse and contrasting samples with cautiously synthesized environmental variables, through a range of diverse last-generation models, offers an unprecedented power for GEA studies in the absence of phenotyping and with moderate sample sizes. By doing this, we identified a broad genetic basis for heat tolerance in common bean, and captured adaptive loci related to the activation of HSPs (*HSFB1*, *MED23*, and *MED25*) as well as HSPs of low molecular weight (*HSP20* and *HSP40*). Small HSP family genes were actually identified as relevant in the recent work by Soltani et al. (2019), where authors detected *HSP21* as one of the three most over-expressed genes in common bean under HS using RNA-sequencing.

On the other hand, the use of traditional GWAS models and raw environmental information should be avoided since they lack statistical power to detect associated markers. Several authors had already pointed this limitation (Cortés and Blair, 2018; Frank et al., 2016; Lasky et al., 2015). Therefore, we suggest coupling synthesized environmental variables with diverse last-generation models, in order to reveal more accurately the adaptive genetic variation to different types of stress in collections of wild germplasm.

## Perspectives

This study demonstrates that the implementation of last-generation GWAS models under a GEA framework with carefully chosen environmental indices improves the reconstruction of the genetic basis of adaptation to HS. New studies across a variety of species and populations subjected to different stresses will benefit by using last-generation GWAS models within a well thought GEA design in order to capture better sources of genetic adaptation. We are looking forward to seeing more studies that follow these lines within the oncoming years.

On the other hand, the genes identified in this study as candidates for heat tolerance have the potential to be used in plant breeding programs after validation by means of strategies such as gene expression studies and Whole Genome re-Sequencing (WGS) (Barbulescu et al., 2018). The latter will make available all the genetic variability present in each accession. Additionally, it would be ideal that the indices explored in this work were contrasted with measurements of heat tolerance in greenhouse and at field conditions under controlled treatments (Zuiderveen et al., 2016). It would also be appropriate to consider for these experiments the same group of accessions used in the present work as well as accessions of related species that are well-known for their heat tolerance (*i.e. Phaseolus acutifolius*). Ultimately, validated candidate genes could be integrated into molecular editing strategies (Lang-Mladek et al., 2010; Pecinka et al., 2010; LeBlanc et al., 2018).

As part of a larger project, promissory accessions identified in this work will be evaluated together with advanced lines and related species under HS conditions at Coastal Colombia. These materials are currently undergoing seed multiplication at the greenhouses so that field establishment can take place in 2020.

Finally, by exploring the genetic basis of heat tolerance using indices constructed from phenotypic information, it will be possible to couple GBS and WGS data with last-generation GWAS models and genomic selection approaches (Crossa et al., 2011). In parallel, there have been recent GWAS developments relying on Artificial Intelligence (AI) (*i.e. deep learning*) and Machine Learning (ML) strategies that deserve further exploration under a GEA framework.

## Data Availability Statement

The filtered dataset and data analysis pipeline are archived at the Dryad Digital Repository (https://doi.org/10.5061/dryad.9k862c8).

## Author Contributions

AC conceived this study. AC carried out DNA extractions to produce GBS data. LL recovered historical environmental data. LL analyzed and interpreted environmental and GBS data with guidance from AC. LL wrote the manuscript with contributions from AC.

## Funding

The genotyping and early analyses done as part of this work were funded by the Lundell and Tullberg grants, with support from the grants 4.J1-2016-00418 and BS2017-0036 from Vetenskapsrådet (VR) and Kungliga Vetenskapsakademien (KVA) to AC as PI. The Geneco Mobility Fund to AC and the Fulbright Specialist Award to M.W. Blair are acknowledged for encouraging synergistic discussions around common bean genetics in Nashville (TN, USA) and Rionegro (Antioquia, Colombia) during 2015 and 2019, respectively. The Network for Vegetable Research and the Training & Development Department from the Colombian Corporation for Agricultural Research are thanked for sponsoring LL’s internship. The Editorial Fund from the same institute is recognized for subsidizing the publication fee of this work.

## Conflict of Interest

The authors declare that the research was conducted in the absence of any commercial or financial relationships that could be construed as a potential conflict of interest.

## Footnotes

1
http://genebank.ciat.cgiar.org/genebank/main.do

2
http://www.worldclim.org

3
https://phytozome.jgi.doe.gov/pz/portal.html

4
http://geneontology.org/

5
https://pfam.xfam.org/

6
http://www.pantherdb.org/

7
https://www.genome.jp/kegg/

8
https://www.uniprot.org/

## Supplementary Material

Table S1 Identity of the 78 common bean accessions used in this study. The G identification number (from the Genetic Resources Unit at the International Center for Tropical Agriculture), country of origin and georeferences. The raw bioclimatic variables used are BIO1 = annual mean temperature, BIO5 = maximum temperature of warmer months, BIO8 = mean temperature of the wettest quarter, BIO9 = mean temperature of the driest quarter, BIO10 = mean temperature of the warmest 4-month period, and *T* *_j_* = average of absolute maximum temperature during the reproductive phase. The bioclimatic-based heat indices are the HSI, HIT, and PCA1. The membership value of 78 common bean accessions using TESS3 algorithm (*K* = 6).

Table S2 List of notations of GEA models generated by last-generation GWAS (SUPER, FarmCPU, and BLINK) and improvement traditional MLM algorithms (CMLM). These notations show, for each model, the family of GWAS models used and algorithms implemented for random and fixed effects. The models were abbreviated as follows: I_M-Fc-Rc_, where “I” refers to the HS index, “M” is the GWAS model family, and “Fc” and “Rc” are the algorithms used to reconstruct the fixed and random covariates, respectively.

Table S3 Genome–environment association (GEA) analyses for heat tolerance according to last-generation GWAS algorithms (SUPER, FarmCPU, and BLINK) for each 270 SNP markers associated with HIT, HSI, and PCA1 indices in 78 common bean accessions based on the optimum association GEA analysis (**Figures 2**–**5**).

Table S4 Summary statistics of last-generation GWAS algorithms (SUPER, FarmCPU, and BLINK). The HS indices and chromosomes (Pv) are presented for each SNP marker associated in 78 common bean accessions based on the optimum association analysis (**Figures 2**–**5**).

Table S5 List of 578 genes flanking 120 associated SNP markers in an expanded genomic window of 81 kb using PhytoMine (see note B) and the reference genome of common bean v2.1

Figure S1 Boxplot, histogram, skewness, kurtosis, and Shapiro–Wilk statistics of the six bioclimatic variables [BIO1 = annual mean temperature **(A)**, BIO5 = maximum temperature of warmer months **(B)**, BIO8 = mean temperature of the wettest quarter **(C)**, BIO9 = mean temperature of the driest quarter **(D)**, BIO10 = mean temperature of the warmest 4-month period **(E)**, and *T* *_j_* = average of absolute maximum temperature during the reproductive phase **(F)**] and the three HS indices [HSI **(G)**, HIT **(H)**, and PCA1 **(I)**] for the 86 common bean accessions used in this study.

Figure S2 Dispersion diagrams generate by means of Pearson **(A)** and Spearman **(B)** correlations for all bioclimatic variables and between each HS index.

Figure S3 Heat maps of kinship matrices estimated with the VanRaden **(A)**, Loiselle **(B)**, and EMMA **(C)** algorithms across all 23,373 SNP markers.

Figure S4 Manhattan and Q–Q plots of the exploratory phase of genome–environment association (GEA) analysis, for heat tolerance in 78 common bean accessions based on 23,373 SNP markers according to traditional MLM algorithm with the population structure as a fixed effect using the first six principal components (**Figure 1D**). Also, these MLM models use kinship matrix as a random effect by means of Loiselle and VanRaden algorithms. These MLM models are HSI_MLM-PC-LOISELLE_ **(A**, **B)**, HIT_MLM-PC-LOISELLE_ **(C**, **D)**, PCA1_MLM-PC-LOISELLE_ **(E**, **F)**, HSI_MLM-PC-_ _VANRADEN_ **(G**, **H)**, HIT_MLM-PC-VANRADEN_ **(I**, **J)**, and PCA1_MLM-PC-VANRADEN_ (**K**, **L**). The blue dashed horizontal line marks the lax *P*-value threshold. The red dots are SNP markers that systematically crossed the lax threshold in the exploratory phase from all 18 MLM models (S1_42870591 in Pv01 and S1_466464831 and S1_471851336 in Pv11). Black and green colors highlight different common bean (Pv) chromosomes.

Figure S5 Manhattan and Q–Q plots of the exploratory phase of genome–environment association (GEA) analysis, for heat tolerance in 78 common bean accessions based on 23,373 SNP markers according to a traditional MLM algorithm with the population structure using TESS3 (**Figure 1F**) as a fixed effect. Also, these MLM models use kinship matrix as a random effect by means of EMMA and Loiselle algorithms. These MLM models are HSI_MLM-TESS3-EMMA_ **(A**, **B)**, HIT_MLM-TESS3-EMMA_ **(C**, **D)**, PCA1_MLM-TESS3-EMMA_ **(E**, **F)**, HSI_MLM-TESS3-LOISELLE_ **(G**, **H)**, HIT_MLM-TESS3-LOISELLE_ **(I**, **J)**, and PCA1_MLM-TESS3-LOISELLE_ **(K**, **L)**. The blue dashed horizontal line marks the lax *P*-value threshold. The red dots are SNP markers that systematically crossed the lax threshold in the exploratory phase from all 18 MLM models (S1_42870591 in Pv01 and S1_466464831 and S1_471851336 in Pv11). Black and green colors highlight different common bean (Pv) chromosomes.

Figure S6 The Manhattan and Q–Q plots of the exploratory phase of genome–environment association (GEA) analysis, for heat tolerance in 78 common bean accessions based on 23,373 SNP markers according to traditional MLM algorithm with population structure as a fixed effect using TESS3 (**Figure 1F**) and kinship matrix as a random effect using the VanRaden algorithm. These MLM models are HSI_MLM-TESS3-VANRADEN_ **(A**, **B)**, HIT_MLM-TESS3-VANRADEN_ **(C**, **D)**, PCA1_MLM-TESS3-VANRADEN_ **(E**, **F)**. The red dots are SNP markers that systematically crossed the lax threshold in the exploratory phase from all 18 MLM models (S1_42870591 in Pv01 and S1_466464831 and S1_471851336 in Pv11). The Manhattan and Q–Q plots of genome–environment association (GEA) analysis, for heat tolerance in 78 common bean accessions based on 23,373 SNP markers according to compressed MLM algorithms with the population structure using TESS3 (**Figure 1F**) as fixed effect and kinship matrix as a random effect using EMMA algorithm. These CMLM models are HSI_CMLM-TESS3-EMMA_ **(G**, **H)**, HIT_CMLM-TESS3-EMMA_ **(I**, **J)**, and PCA1_CMLM-TESS3-EMMA_ **(K**, **L)**. The blue dashed horizontal line marks the lax *P*-value threshold. Black and green colors highlight different common bean (Pv) chromosomes.

Figure S7 Manhattan and Q–Q plots of genome–environment association (GEA) analysis by means of SUPER algorithm, for heat tolerance in 78 common bean accessions based on 23,373 SNP. GLM model is used in the first step of these nine “failed” SUPER models, and the last step used CMLM (A–F) and MLM (G–P) algorithms. The nine “failed” SUPER are HSISUPER(CMLM)- TESS3-EMMA (A, B), HITSUPER(CMLM)-TESS3-EMMA (C, D), PCA1SUPER(CMLM)-TESS3-EMMA (E, F), HSISUPER(MLM)-PC-EMMA (G, H), HITSUPER(MLM)-PC-EMMA (I, J), PCA1SUPER(MLM)-PC-EMMA (K, L), HSISUPER(MLM)-TESS3-EMMA (M, N), HITSUPER(MLM)-TESS3-EMMA (O, P), and PCA1SUPER(MLM)-TESS3-EMMA (Q, R). Black and green colors highlight different common bean (Pv) chromosomes.

Figure S8 Historical maximum temperature values obtained from the monthly averages from years 1970 to 2000. Extraction of information from WorldClim (see note B). Map construction was done through a customized R-Script using the *raster* package of R v. 3.6.1 (R Core Team).
