# Supplementary figures and images for: Last-Generation Genome–Environment Associations Reveal the Genetic Basis of Heat Tolerance in Common Bean (Phaseolus vulgaris L.)
Source: Front Genet. 2019 Nov 22;10:954. doi: 10.3389/fgene.2019.00954 (PMC6883007; doi:10.3389/fgene.2019.00954)

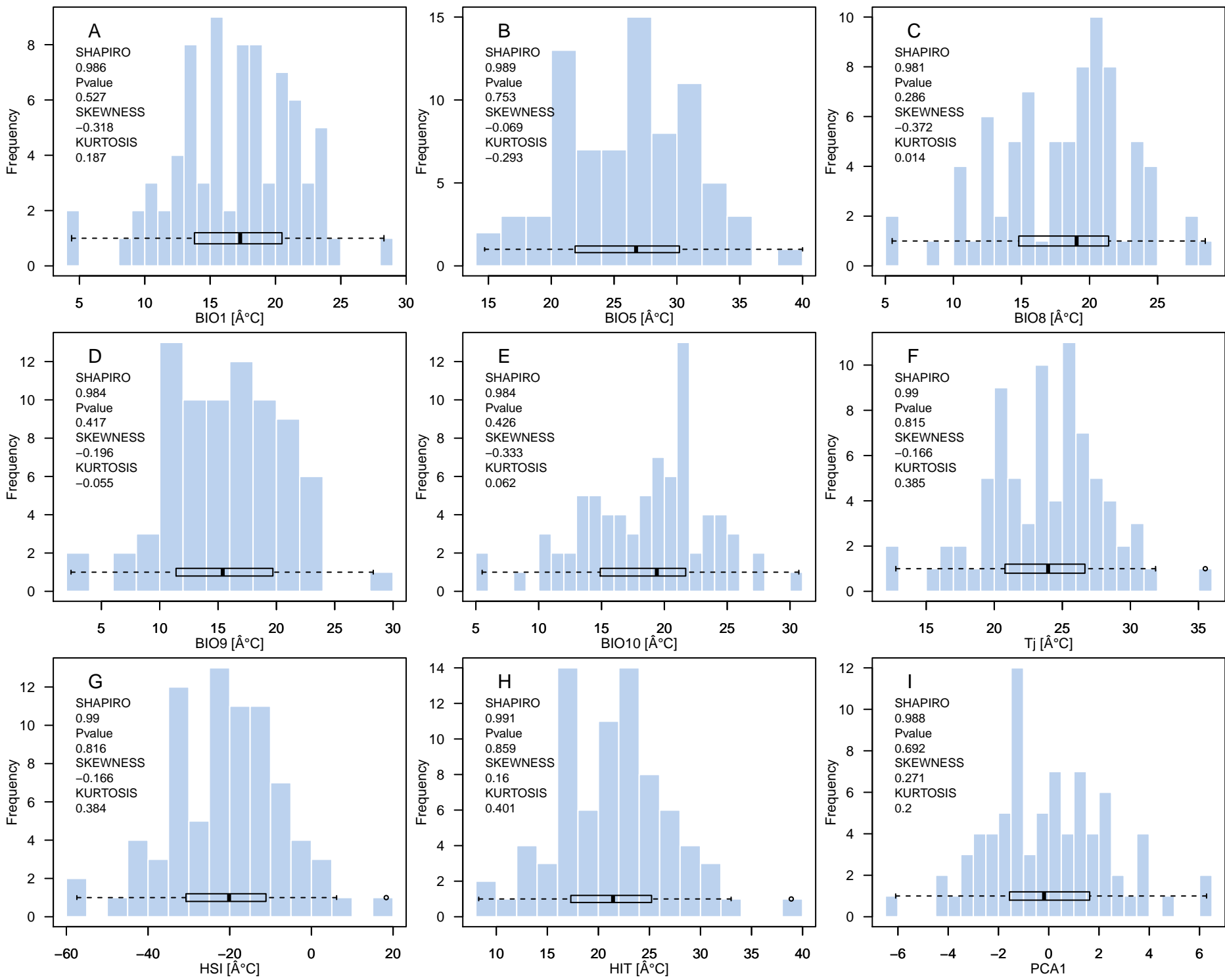

Supplement: Figure S1 — Boxplot, histogram, skewness, kurtosis, and Shapiro–Wilk statistics of the six bioclimatic variables [BIO1 = annual mean temperature (A), BIO5 = maximum temperature of warmer months (B), BIO8 = mean temperature of the wettest quarter (C), BIO9 = mean temperature of the driest quarter (D), BIO10 = mean temperature of the warmest 4-month period (E), and T j = average of absolute maximum temperature during the reproductive phase (F)] and the three HS indices [HSI (G), HIT (H), and PCA1 (I)] for the 86 common bean accessions used in this study. [file DataSheet_1.pdf]

A

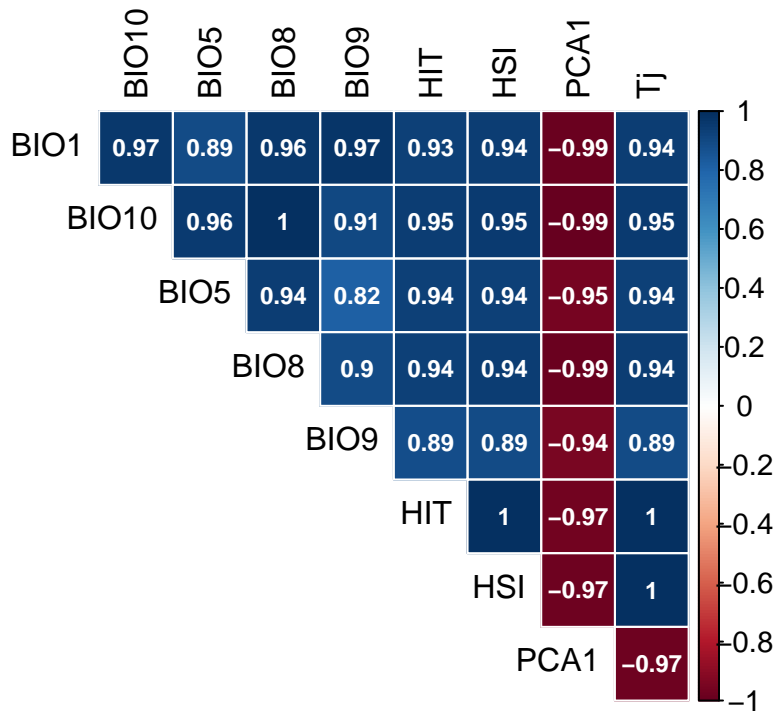

B

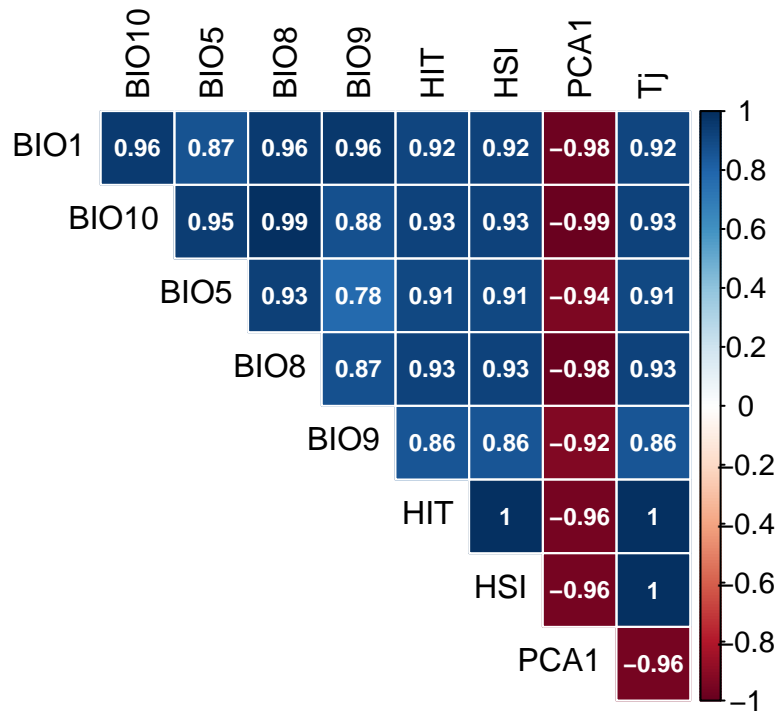

Supplement: Figure S2 — Dispersion diagrams generate by means of Pearson (A) and Spearman (B) correlations for all bioclimatic variables and between each HS index. [file DataSheet_2.pdf]

œ

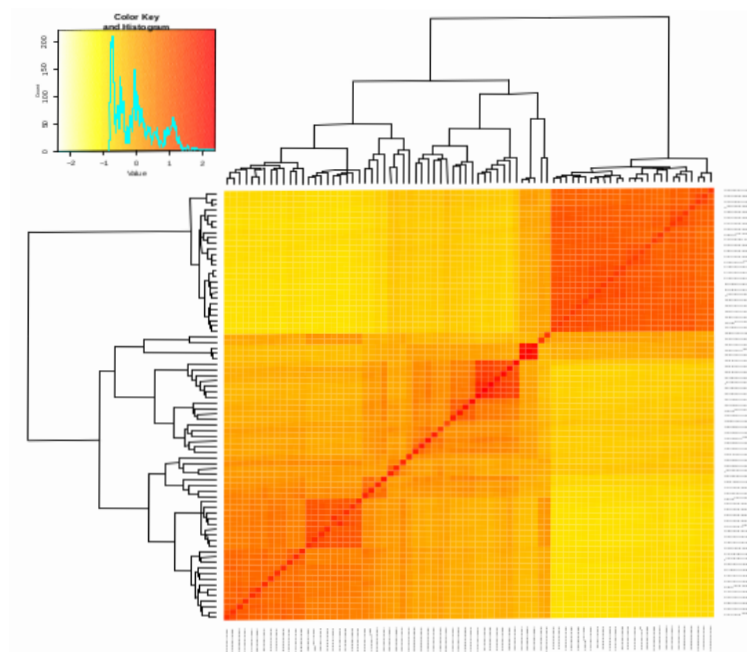

ó

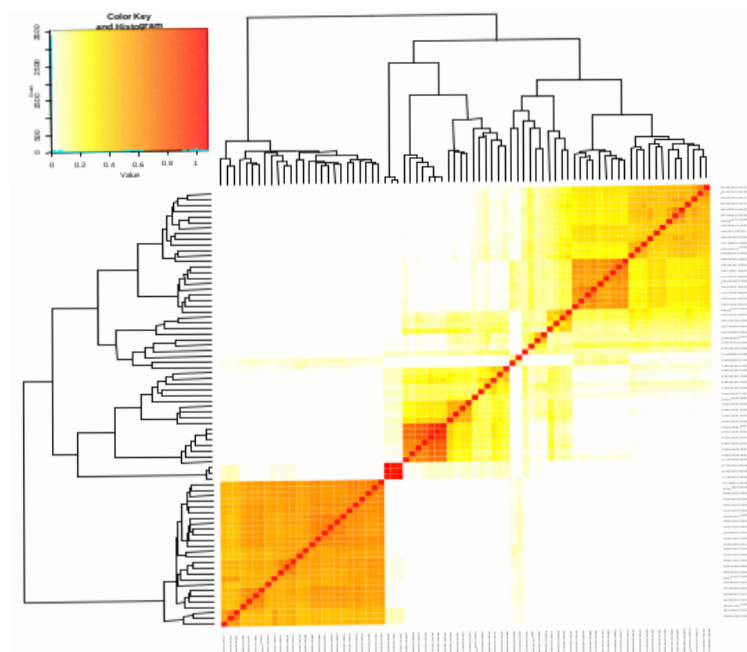

ô

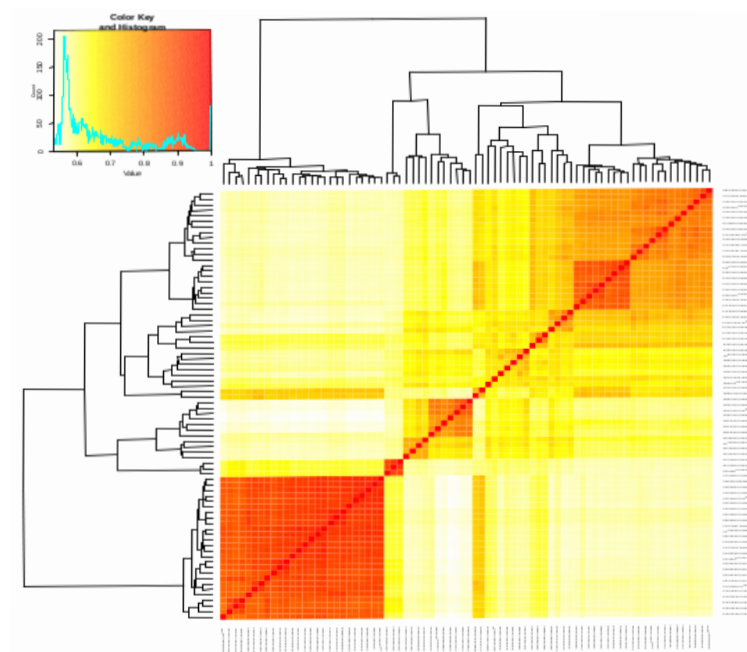

Supplement: Figure S3 — Heat maps of kinship matrices estimated with the VanRaden (A), Loiselle (B), and EMMA (C) algorithms across all 23,373 SNP markers. [file DataSheet_3.pdf]

Observed  $-\log(\text{Pvalue})$

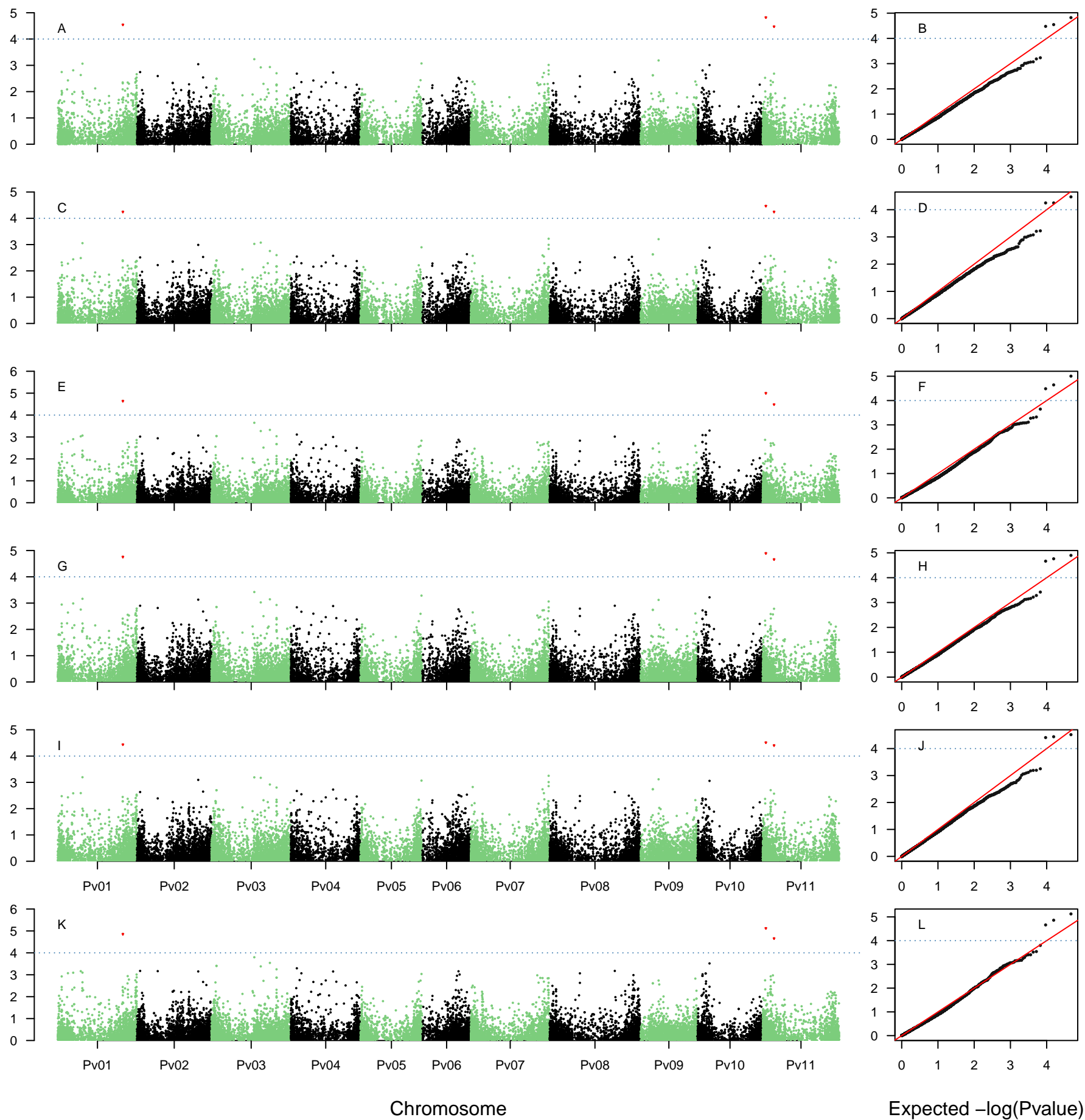

Supplement: Figure S4 — Manhattan and Q–Q plots of the exploratory phase of genome–environment association (GEA) analysis, for heat tolerance in 78 common bean accessions based on 23,373 SNP markers according to traditional MLM algorithm with the population structure as a fixed effect using the first six principal components ( Figure 1D ). Also, these MLM models use kinship matrix as a random effect by means of Loiselle and VanRaden algorithms. These MLM models are HSIMLM-PC-LOISELLE (A, B), HITMLM-PC-LOISELLE (C, D), PCA1MLM-PC-LOISELLE (E, F), HSIMLM-PC- VANRADEN (G, H), HITMLM-PC-VANRADEN (I, J), and PCA1MLM-PC-VANRADEN (K, L). The blue dashed horizontal line marks the lax P-value threshold. The red dots are SNP markers that systematically crossed the lax threshold in the exploratory phase from all 18 MLM models (S1_42870591 in Pv01 and S1_466464831 and S1_471851336 in Pv11). Black and green colors highlight different common bean (Pv) chromosomes. [file DataSheet_4.pdf]

Observed  $-\log(\text{Pvalue})$

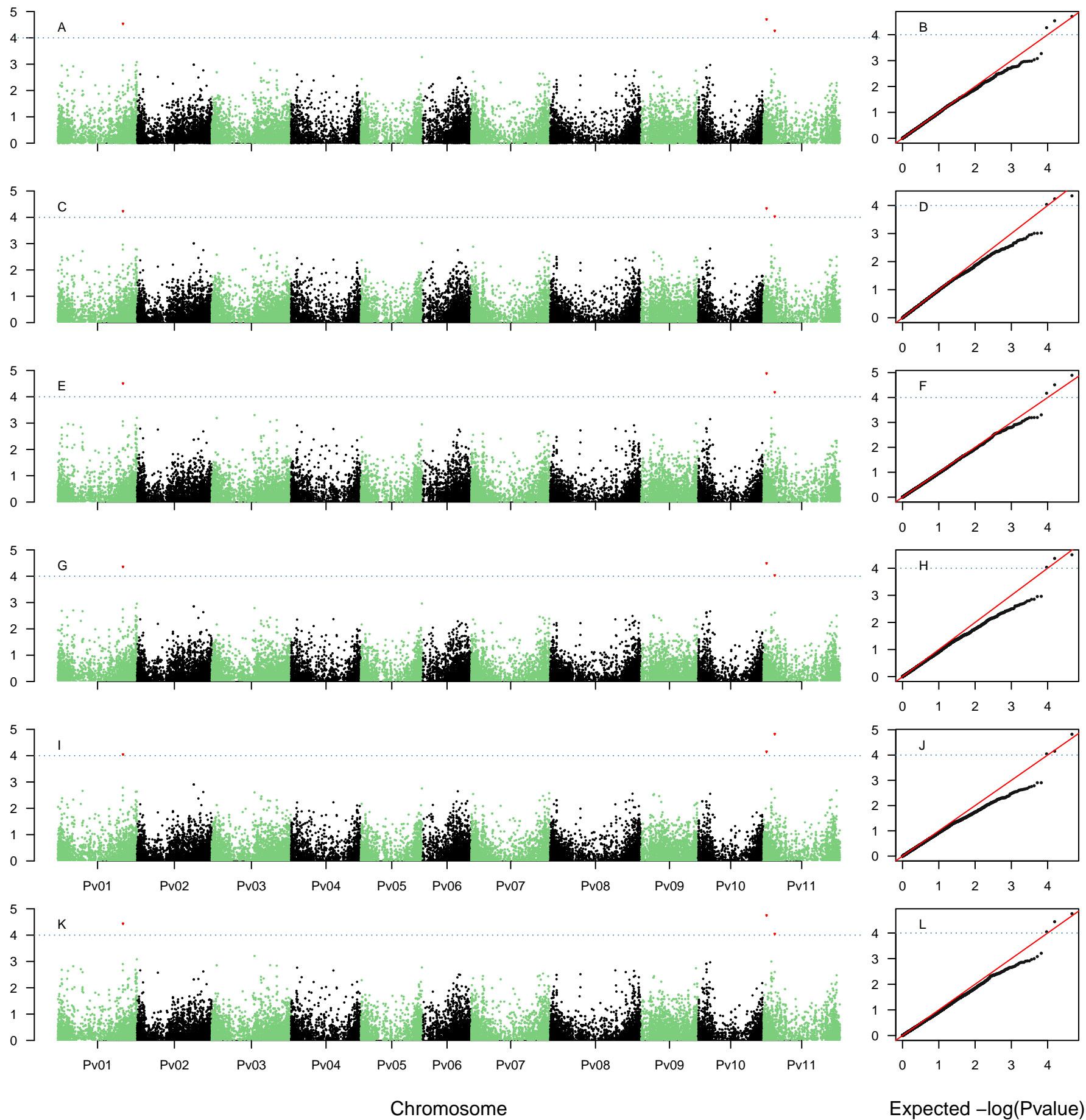

Supplement: Figure S5 — Manhattan and Q–Q plots of the exploratory phase of genome–environment association (GEA) analysis, for heat tolerance in 78 common bean accessions based on 23,373 SNP markers according to a traditional MLM algorithm with the population structure using TESS3 ( Figure 1F ) as a fixed effect. Also, these MLM models use kinship matrix as a random effect by means of EMMA and Loiselle algorithms. These MLM models are HSIMLM-TESS3-EMMA (A, B), HITMLM-TESS3-EMMA (C, D), PCA1MLM-TESS3-EMMA (E, F), HSIMLM-TESS3-LOISELLE (G, H), HITMLM-TESS3-LOISELLE (I, J), and PCA1MLM-TESS3-LOISELLE (K, L). The blue dashed horizontal line marks the lax P-value threshold. The red dots are SNP markers that systematically crossed the lax threshold in the exploratory phase from all 18 MLM models (S1_42870591 in Pv01 and S1_466464831 and S1_471851336 in Pv11). Black and green colors highlight different common bean (Pv) chromosomes. [file DataSheet_5.pdf]

Observed  $-\log(\text{Pvalue})$

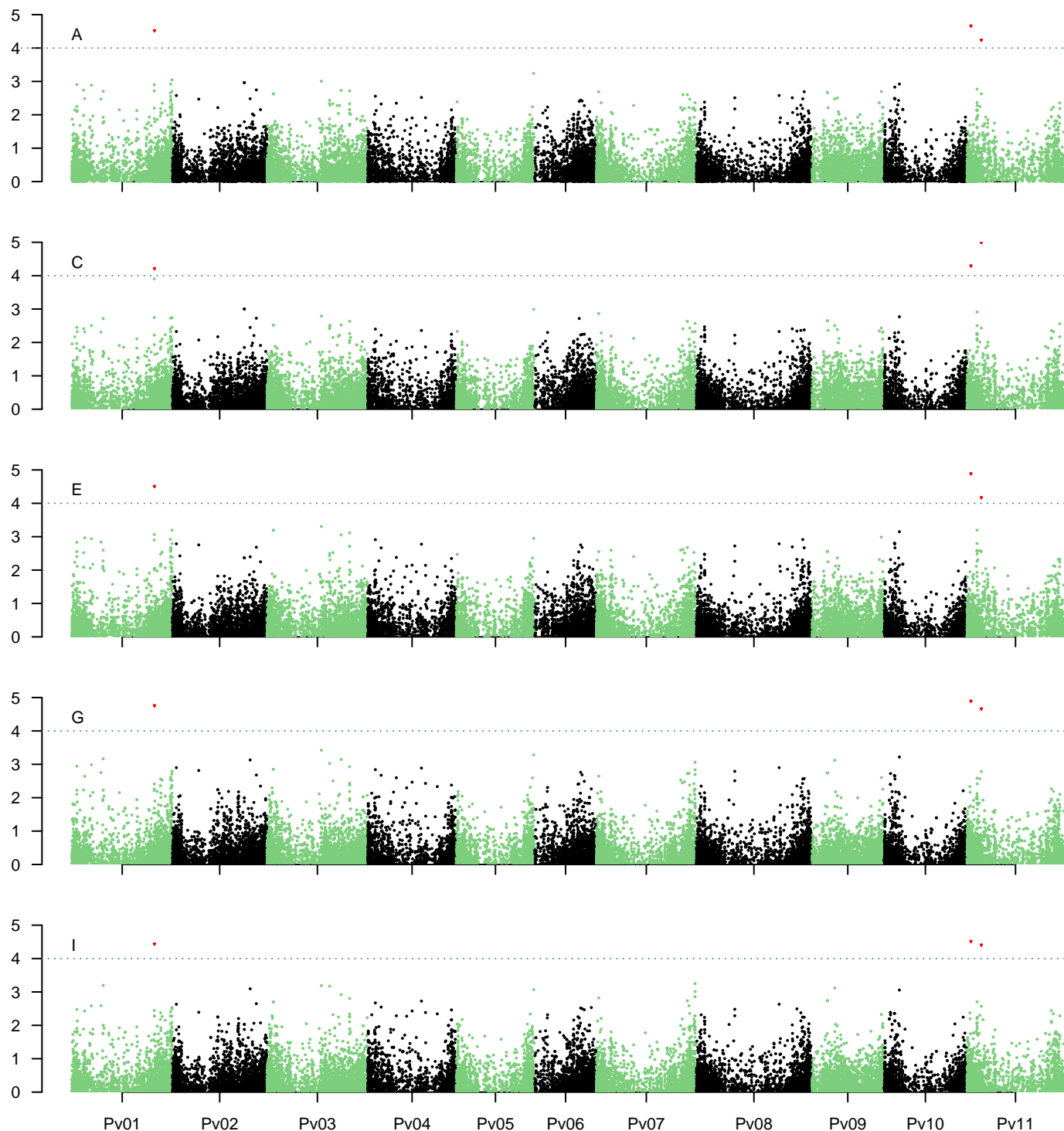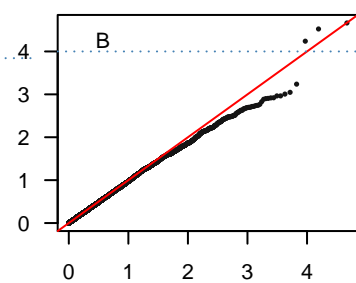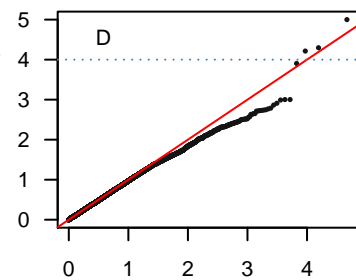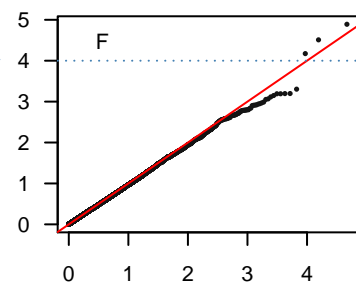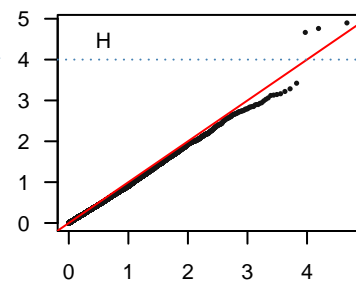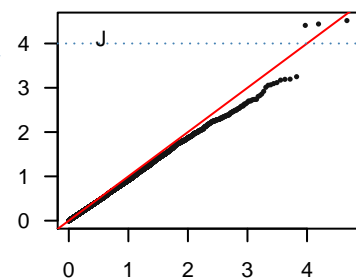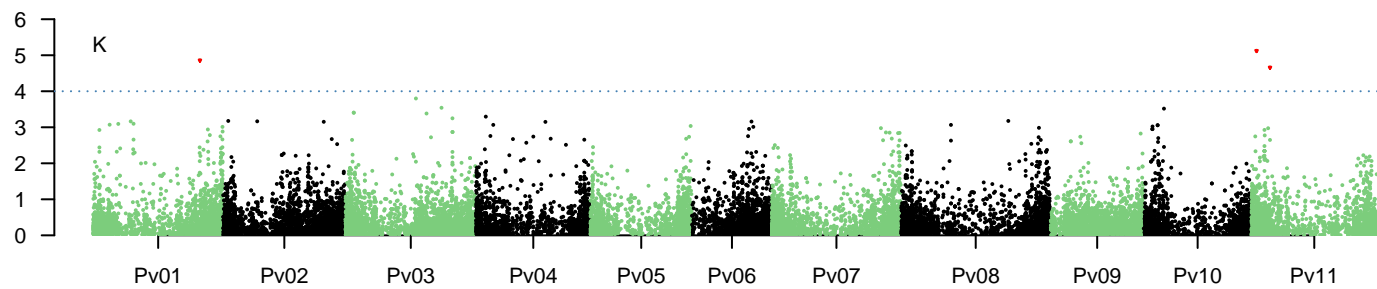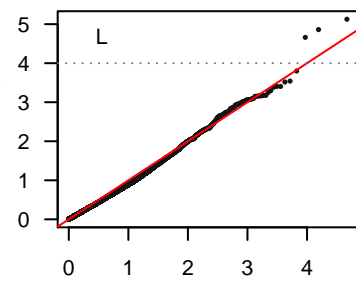

Chromosome

Expected  $-\log(\text{Pvalue})$

Supplement: Figure S6 — The Manhattan and Q–Q plots of the exploratory phase of genome–environment association (GEA) analysis, for heat tolerance in 78 common bean accessions based on 23,373 SNP markers according to traditional MLM algorithm with population structure as a fixed effect using TESS3 ( Figure 1F ) and kinship matrix as a random effect using the VanRaden algorithm. These MLM models are HSIMLM-TESS3-VANRADEN (A, B), HITMLM-TESS3-VANRADEN (C, D), PCA1MLM-TESS3-VANRADEN (E, F). The red dots are SNP markers that systematically crossed the lax threshold in the exploratory phase from all 18 MLM models (S1_42870591 in Pv01 and S1_466464831 and S1_471851336 in Pv11). The Manhattan and Q–Q plots of genome–environment association (GEA) analysis, for heat tolerance in 78 common bean accessions based on 23,373 SNP markers according to compressed MLM algorithms with the population structure using TESS3 ( Figure 1F ) as fixed effect and kinship matrix as a random effect using EMMA algorithm. These CMLM models are HSICMLM-TESS3-EMMA (G, H), HITCMLM-TESS3-EMMA (I, J), and PCA1CMLM-TESS3-EMMA (K, L). The blue dashed horizontal line marks the lax P-value threshold. Black and green colors highlight different common bean (Pv) chromosomes. [file DataSheet_6.pdf]

Observed  $-\log(\text{Pvalue})$

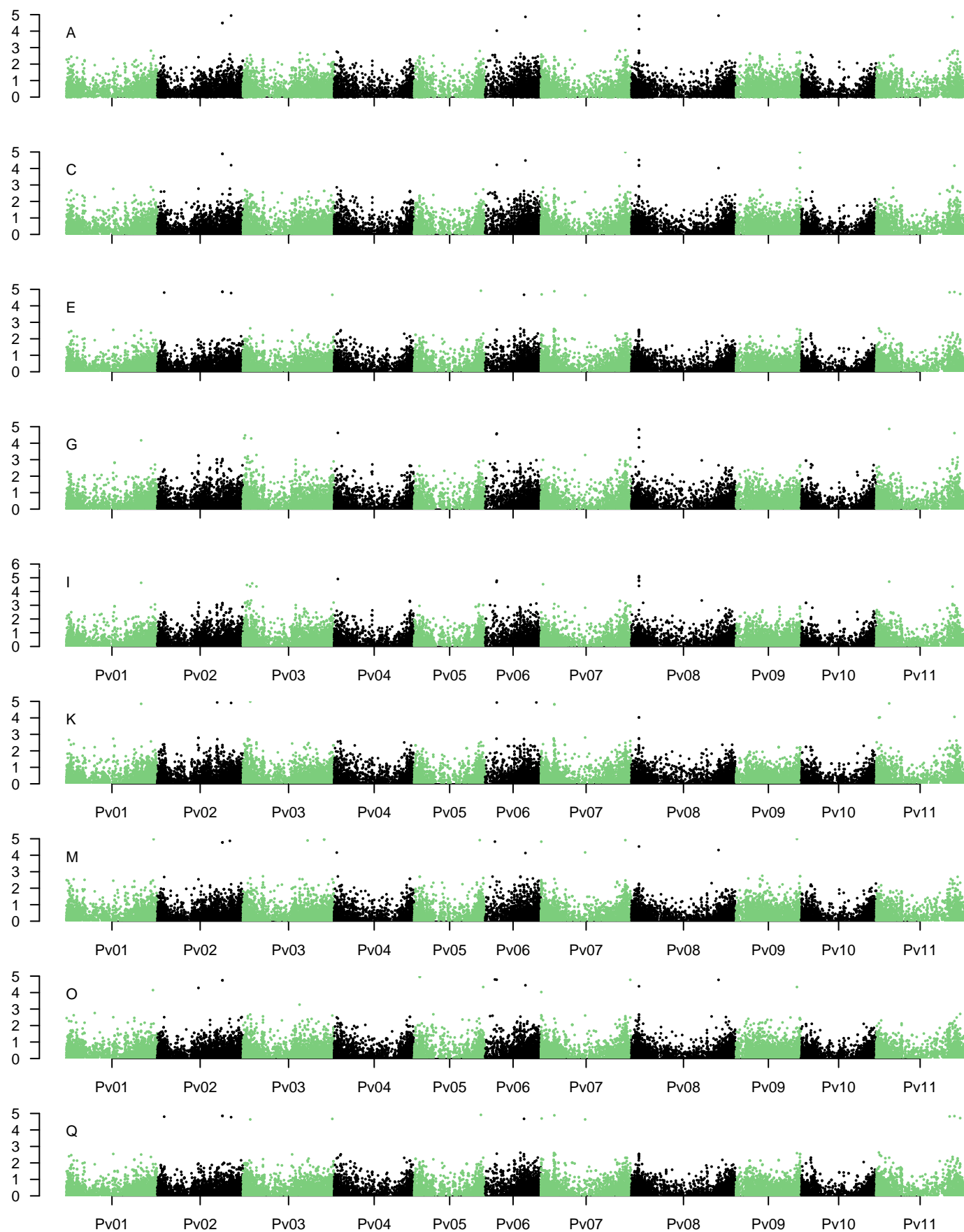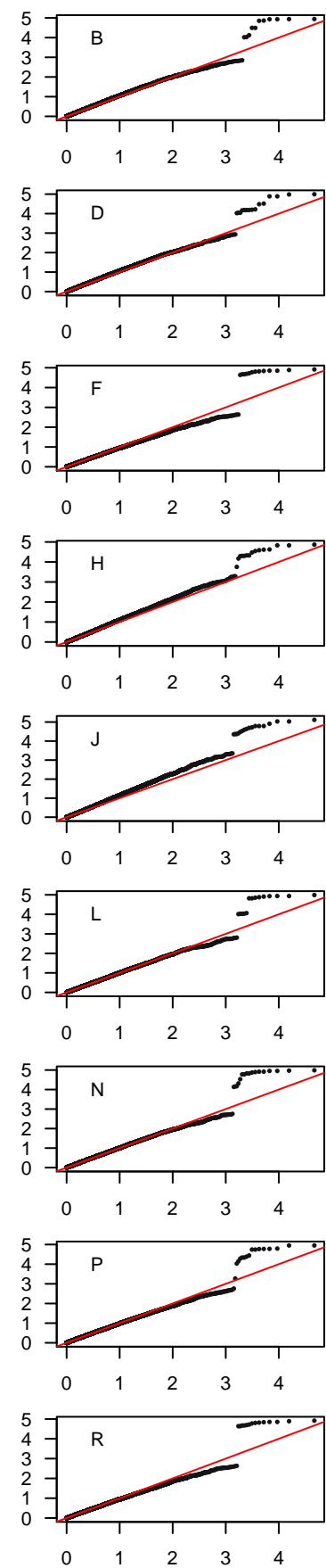

Supplement: Figure S7 — Manhattan and Q–Q plots of genome–environment association (GEA) analysis by means of SUPER algorithm, for heat tolerance in 78 common bean accessions based on 23,373 SNP. GLM model is used in the first step of these nine “failed” SUPER models, and the last step used CMLM (A–F) and MLM (G–P) algorithms. The nine “failed” SUPER are HSISUPER(CMLM)- TESS3-EMMA (A, B), HITSUPER(CMLM)-TESS3-EMMA (C, D), PCA1SUPER(CMLM)-TESS3-EMMA (E, F), HSISUPER(MLM)-PC-EMMA (G, H), HITSUPER(MLM)-PC-EMMA (I, J), PCA1SUPER(MLM)-PC-EMMA (K, L), HSISUPER(MLM)-TESS3-EMMA (M, N), HITSUPER(MLM)-TESS3-EMMA (O, P), and PCA1SUPER(MLM)-TESS3-EMMA (Q, R). Black and green colors highlight different common bean (Pv) chromosomes. [file DataSheet_7.pdf]
