# Supplementary material for: Last-Generation Genome–Environment Associations Reveal the Genetic Basis of Heat Tolerance in Common Bean (Phaseolus vulgaris L.)
Source: Front Genet. 2019 Nov 22;10:954. doi: 10.3389/fgene.2019.00954 (PMC6883007; doi:10.3389/fgene.2019.00954)
Supplement: Figure S8 — Historical maximum temperature values obtained from the monthly averages from years 1970 to 2000. Extraction of information from WorldClim (see note B). Map construction was done through a customized R-Script using the raster package of R v. 3.6.1 (R Core Team). [file DataSheet_8.pdf]

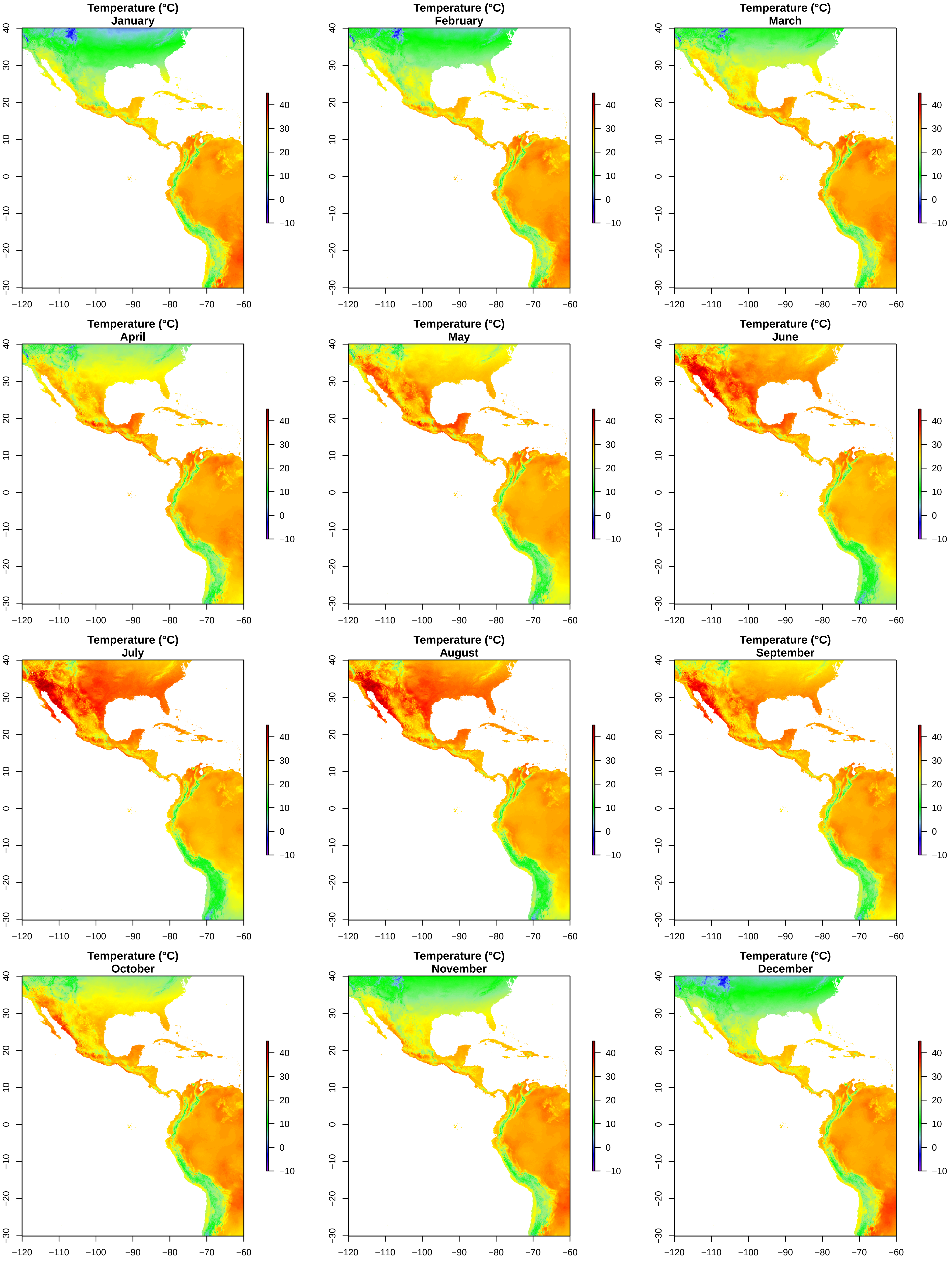
Temperature (°C)
January
Temperature (°C)
February
Temperature (°C)
March
Temperature (°C)
April
Temperature (°C)
May
Temperature (°C)
June
Temperature (°C)
July
Temperature (°C)
August
Temperature (°C)
September
Temperature (°C)
October
Temperature (°C)
November
Temperature (°C)
December
40
30
20
10
0
−10
−20
−30
−120
−110
−100
−90
−80
−70
−60
